# Genome-Wide Association Study of Healthful Flavonoids among Diverse Mandarin Accessions

**DOI:** 10.3390/plants11030317

**Published:** 2022-01-25

**Authors:** Matthew R. Mattia, Dongliang Du, Qibin Yu, Tracy Kahn, Mikeal Roose, Yoko Hiraoka, Yu Wang, Patricio Munoz, Fred G. Gmitter

**Affiliations:** 1 Citrus Research and Education Center, Department of Horticultural Sciences, University of Florida, Lake Alfred, FL 33850, USA; mrmattia17@gmail.com (M.R.M.); learnholickyle@gmail.com (D.D.); qibin@ufl.edu (Q.Y.); yu.wang@ufl.edu (Y.W.); 2 Department of Botany and Plant Sciences, University of California, Riverside, CA 92521, USA; tracy.kahn@ucr.edu (T.K.); mikeal.roose@ucr.edu (M.R.); yoko.eck@ucr.edu (Y.H.); 3 Department of Horticultural Sciences, University of Florida, Gainesville, FL 32611, USA; p.munoz@ufl.edu

**Keywords:** citrus, antioxidant, GWAS, LCMS, plant breeding, candidate genes, phenotyping, polyphenols

## Abstract

Mandarins have many unique flavonoids with documented health benefits and that help to prevent chronic human diseases. Flavonoids are difficult to measure and cannot be phenotyped without the use of specialized equipment; consequently, citrus breeders have not used flavonoid contents as selection criteria to develop cultivars with increased benefits for human health or increased tolerance to diseases. In this study, peel, pulp, and seed samples collected from many mandarin accessions and their hybrids were analyzed for the presence of selected flavonoids with documented human health benefits. A genome-wide association study (GWAS) was used to identify SNPs associated with biosynthesis of flavonoids in these mandarin accessions, and there were 420 significant SNPs were found to be associated with 28 compounds in peel, pulp, or seed samples. Four candidate genes involved in flavonoid biosynthesis were identified by enrichment analysis. SNPs that were found to be associated with compounds in pulp samples have the potential to be used as markers to select mandarins with improved phytonutrient content to benefit human health. Mandarin cultivars bred with increased flavonoid content may provide value to growers and consumers.

## 1. Introduction

Beneficial flavonoid content may be a consumer-driven trait that potentially can be improved through breeding and used as a tool to differentiate mandarins with convenience traits in the marketplace, as mandarins with higher levels of healthful flavonoids and marketed as such may be preferred by health-conscious consumers. Flavonoids are a diverse, large group of plant-based compounds, many of which are reported to have beneficial health effects. Consumers are seeking foods that improve their quality of life and prevent nutrition-dependent chronic diseases [1]. Citrus contains many unique flavonoids known to have health-promoting properties, specifically monoterpenes and flavanones, which are rarely present in other plants [2]. Mandarins are a large and phenotypically diverse group of citrus with a flavonoid composition that has been analyzed in “wild Chinese” [3] and conventional cultivars [4]. A recent study by Wang et al. [5], showed the metabolic diversity of flavonoids in citrus species, including 14 mandarin cultivars that were tested to showcase flavonoid diversity. Differences in mandarin flavonoid contents suggests a genetic component that can be exploited in a modern plant breeding program.

Citrus breeding is a long-term process and has many complexities that other crop species do not. Citrus has a long juvenility period and large tree size, which can substantially increase cultivar release time and the costs associated with running a breeding program. The University of Florida Citrus Research and Education Center (UF-CREC) has an extensive mandarin breeding program that focuses on the development of seedless, flavorful, attractive, easy peeling, and disease resistant cultivars [6]. The use of genetic technologies and their implementation in modern plant breeding programs such as association mapping (AM) and high-throughput genotyping can reduce breeding program costs, shorten cultivar release time, and be used to track genes controlling difficult-to-measure traits [7]. AM takes advantage of the inheritance of functional polymorphisms, historical recombination, and natural genetic diversity to establish linkage disequilibrium (LD) between a gene controlling the trait and a molecular marker used in the association with the trait. There have been many AM studies in plants with short life cycles due to the availability of large amounts of genetic information, namely single nucleotide polymorphisms (SNPs) [8].

The advent of less expensive sequencing technologies, reference genome sequences, and high-throughput methodology has led to an advanced form of AM called genome-wide association studies (GWAS). GWAS uses large numbers of annotated SNPs covering most of the genome, and a large number of genotyped individuals. GWAS has been successful in identifying significant associations for important traits in many agronomic crops [9,10,11]. Implementation of GWAS is an additional tool that can be used to improve the efficiency of fruit tree breeding [12]. Researchers in fruit tree crops have successfully started to apply AM technologies and GWAS in breeding programs. In a peach population of 100 landraces, QTLs were found by AM for flesh color, texture, and other traits of agronomic importance [13]. In apple, significant associations were found for fruit firmness and weight [14]. Mapping grape fruit color using GWAS has successfully identified an associated QTL [15]. GWAS with a small number of SNPs (1841) was used to identify genomic regions associated with fruit quality traits in 111 citrus individuals [16].

A general trend in plant breeding has been observed that the nutritional value of crops, including flavonoids, decreases as yield has increased due to the strong selection pressure for yield [17]. Decreased nutritional value also may have inadvertently occurred within mandarin germplasm, in the absence of deliberate selection for flavonoid content. Plant breeders can use GWAS tools to associate flavonoid traits to specific regions of the genome, to identifying genetic loci and developing markers to select for traits that are difficult to measure. The information obtained from GWAS can be used to make the breeding process more efficient [18]. GWAS can identify molecular markers to be used to select mandarin cultivars with improved phytonutrient content and human health benefits. Additionally, high flavonoid accumulation has been associated with a reduction of diseases in many plant species [19]. Thus, mandarins with increased flavonoid accumulation can be part of the solution to developing cultivars with better disease and pest resistances.

The objective of this study was to identify SNPs and candidate genes associated with flavonoid content using a GWAS approach. To achieve our objective, flavonoid compounds (28) were quantified in mandarin peel, pulp, and seed samples in a population of 137 diverse mandarin, mandarin hybrid, and ancestral accessions from the University of California, Riverside’s Citrus Variety Collection (UCR-CVC). Our hypothesis was that we would find genetic associations with flavonoids and identify candidate genes through enrichment analysis that could be used to potentially develop markers to breed for mandarins with higher levels of healthful flavonoids.

## 2. Materials and Methods

### 2.1. Mapping Population

The mapping population consisted of 137 accessions of mandarin and mandarin hybrids (Table S1, Diverse mandarin accessions used for the genome wide association of healthful flavonoids). Six fruits for each accession were collected at the UCR-CVC in December of 2016. Fruits were washed, packaged in food safe plastic bags and shipped in crates to Florida within 2 days of harvest for sample preparation and analysis.

### 2.2. Chemical Reagents and Standard Compounds

Chromographic-grade acetonitrile, formic acid, methanol, and HPLC grade water were purchased from Thermo Fisher (Hanover Park, IL, USA). Analytical-grade standard compounds apigenin, coumarin, didymin, diosmetin, diosmin, eriocitrin, eriodictyol, heptamethoxyflavone, hesperetin, hesperidin, isosakurenetin, isosinensetin, kaempferol, limonin, luteolin, naringenin, naringin, narirutin, neodiosmin, neoeriocitrin, neohesperidin, nobiletin, nomilin, poncirin, quercetin, rhoifolin, rutin, scopoletin, scutellarein, sinensetin, tangeretin, taxafolin, umbelliferone, and the internal standard catechin were purchased from Indofine Chemical (Hillsborough, NJ, USA). All flavonoid standards were dissolved in methanol and stored at −80 °C.

### 2.3. Sample Preparation and Flavonoid Extraction

Peel (flavedo and albedo), pulp (juice vesicles, segments, and segments walls) and seeds (if present) were collected from the whole fruit. Peel and pulp samples were collected for each accession as follows: the fruits were divided into three equal groups that contained at least two fruit per biological replicate. Peel and pulp were separated and ground into a fine powder with liquid nitrogen and stored at −80 °C. Seeds were bulked per accession and separated into three experimental replicates and ground with liquid nitrogen and stored at −80 °C. Peel, pulp and seed samples were freeze dried with a Lab Conoco Freezone 2.5 L freeze dryer (Kansas City, MO, USA) for five days until completely dry. Approximately ten milligrams of sample were weighed into 2 mL centrifuge tubes and exact weights were recorded for dry weight quantification. The extraction was done according to De Vos et al. [20]. Briefly, 1 mL of a solution of 0.1% formic acid in methanol was added to 10 mg of dry sample. Samples were vortexed and sonicated then centrifuged and dried in a Thermo Fisher Speed Vac Concentrator (Hanover Park, IL, USA). The dry extract was reconstituted in 1 mL of methanol and purified with SPE C18 cartridges from Restek (Bellefonte, PA, USA) and filtered with 0.22 μm, 13 mm diameter nylon syringe filters. Filtered extracts were transferred to LC-MS analytical vials and stored at −80 °C until analysis.

### 2.4. Analysis of Flavonoids with LC-MS/MS and Peak Detection and Quantification

Flavonoid analysis was performed with a Thermo TSQ Quantum Ultra triple quadrupole electrospray ionization coupled with a mass spectrometer, with an Accela 1250 quaternary UHPLC pump (LC-MS/MS) (Waltham, MA, USA). The system was equipped with an autosampler, a drawer kept at 10 °C and a nitrogen degasser. Flavonoids were separated on a Phenomonex Gemini C18 reverse phase column (3 μm 150 × 3 mm; Torrance, CA, USA). The column was kept at room temperature through the entire program. The gradient elution program was arranged with two eluants: eluant A, 0.1% formic acid in HPLC water, and eluant B, 0.1% formic acid in acetonitrile. The injection volume was 5 μL. The 45-min program was as follows: 0–20 min, 5–75% B, 25–26 min, ramped to 95% B; 26–33 min, 95% B with a flow of 0.2 mL min^−1^. The mass spectrometry parameters were set as follows: spray voltage 3500 V (positive mode) and 2500 V (negative mode), sheath gas at 45 Arb, aux gas at 20 Arb, sweep gas at 1 Arb; CID gas at 1.5 mTorr, ion transfer tube temperature set at 235 °C, and vaporizer temperature set at 275 °C.

Flavonoid standards were infused individually into the mass spectrophotometer system to determine the correct *m*/*z* values for product ions. Standards were injected individually into the LC-MS/MS system to determine correct retention times. Retention times and *m*/*z* values were input into the Trace Finder software system (Thermo Scientific) for semi-automatic identification of compounds in samples. Standard mixtures of known concentrations were injected ten times to determine limits of detection (LOD) and limits of quantification (LOQ). Each compound was considered to be detected at 3:1 and quantified at a 10:1 signal to noise ratio [21]. Flavonoids in samples were quantified as the area under the curve relative to the area under the curve of the internal standard catechin. The quantification was calculated by dividing the relative area by the recorded dry weight for each sample, giving micrograms of compound per milligram of dry weight sample.

### 2.5. GWAS

**Figure 1:**
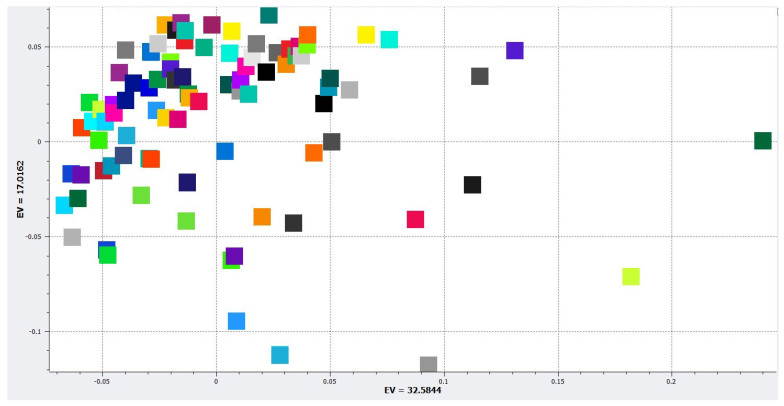
PCA determined population structure for individuals used in the GWAS analysis. Each colored square represents an individual citrus accession.

High-density SNP genotype data was generated with Axiom^TM^ Citrus 56AX (Affymetrix, Inc., Santa Clara, CA, USA) (58 K autosomal and 500 Chloroplast SNPs) developed by University of California, Riverside [22]. Stringent PolyHighResolution (PHR) loci were classified by Axiom^TM^ Analysis Suite v1.1.1.66 (Affymetrix, Santa Clara, CA, USA). The genotyping data were composed of a total number of 51,297 nuclear SNPs distributed across all chromosomes. SNPs were filtered by call rate and minor allele frequency and linkage disequilibrium (LD) pruning using SNP and Variation Suite (SVS) V_8.6.0 (SVS, Golden Helix, Inc., Bozeman, MT, USA, http://www.goldenhelix.com (accessed on 17 July 2018)). The total number of SNPs was filtered with a call rate (CR > 0.95) and a minor allele frequency (MAF > 0.05) to yield 21,451 SNPs. The MAF and CR filtered data were LD pruned with a threshold of 0.5 calculated with the composite haplotype method (CHM) and data were LD pruned leaving 15,913 SNPs with low pairwise LD [23]. Population structure was determined using principal component analysis (PCA; Figure 1). An identity by descent (IBD) kinship matrix was created using Efficient Mixed-Model.

Association Expedited (EMMAX) [24].

The association analysis was performed for each flavonoid compound measured in each fruit sample using the Clementine mandarin genome v1.0 as a reference (https://phytozome.jgi.doe.gov (accessed on 17 July 2018)). Twenty-eight of the 32 compounds that were chosen for GWAS were successfully quantified in mandarin accessions. Using the software SNP and Variation Suite (SVS), a mixed linear model (MLM) was used to make associations and population structure was corrected using a kinship matrix and PCA as covariates in MLM model. Manhattan plots were created using the −log10 *p* values for all LD-pruned SNPs used in the study. Q-Q plots were produced by plotting “expected −log10 *p* values” on the x-axis and “observed −log10 *p* values” on the y-axis. The effective number of independent SNPs was calculated using the simple m software (http://simplem.sourceforge.net (accessed on 17 July 2018)) [25] in R (R Core Team 2018). Significant associations were made when false discovery rate (FDR) was less than 1.23 × 10^−5^ or −log10 (*p* values) were greater than 4.9. Subsequently, a sub-network enrichment analysis (SNEA) in Pathway Studio [26] was done using the default settings. SNEA uses a global expression regulatory network extracted from the entire PubMed database and full-text journals to extract regulatory networks. Using the non-parametric Mann-Whitney test, SNEA identified significant (*p* ≤ 0.05), or over-represented (*p* ≤ 0.05) ontologies. The SNEA determined homologous genes and their specific involvement in biological processes. Genes were considered candidates if annotations were identified as involved in flavonoid related biological processes.

## 3. Results

### 3.1. Analysis of 28 Target Flavonoids among Diverse Mandarin Accessions

Twenty-eight target compounds were quantified in at least one sample type of the mandarin accessions, and 25 compounds were quantified in all samples (Table 1). The compounds quercetin, rhoifolin, taxifolin, and scutellarein were not detected in any mandarin accession. The mean, minimum, and maximum concentrations of each compound varied depending on the compound and the sample (Table 1). The minimum value zero was considered to be below the detection limit for the LC-MS/MS instrument. Most compounds had minimum values below the detection limit represented by a 0 µg/g value (Table 1). Coumarin was not detected in seed samples for any accession. Kaempferol was only detected in seed samples, while umbelliferone was only detected in peel samples. Hesperidin, nobiletin, and tangeretin were detected in all accessions and samples types indicated by minimum values greater than 0 µg/g (Table 1). Neodiosmin was detected in seed of all accessions (min = 0.030 µg/g) but was not detected in peel and pulp samples of some accessions (Table 1). Limonin and heptamethoxyflavone were detected in pulp and seed of all accessions but were below detectable levels for some accessions in peel samples. Didymin was detected in pulp of all accessions (min = 0.0050 µg/g) but had below detectable levels for peel and seed in some accessions (Table 1).

### 3.2. GWAS

**Figure 2:**
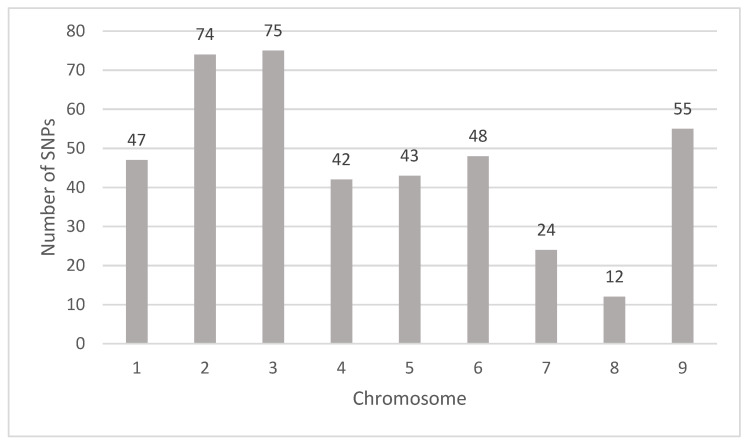
Numbers of significant SNPSs identified for all analyzed samples and components by citrus chromosome using the Clementine mandarin genome v1.0 as a reference.

GWAS was performed with a subset of SNPs that were LD pruned and filtered with a CR > 0.9 and MAF> 0.05, thus generating 15,913 SNPs that were used for analysis. GWAS was performed for each compound detected in each sample resulting in a total of 420 SNPs (Table S2, List of GWAS determined SNPs in mandarin peel, pulp, and seed samples). In total, fifty-three Manhattan plots showing significant SNPs were generated with Q-Q plots that show model fitness (Figure S1, Genome Wide Association Manhattan and Quantile-Quantile Plots Presented by Compound for Significant SNPS). Significant SNPs were found on all nine chromosomes with the greatest number (75) of SNPs located on chromosome 3, and the next greatest on chromosome 2 (74) (Figure 2). Chromosome 8 had the least number (12) of significant SNPs (Figure 2). No significant associations were found for the compounds neodiosmin, rutin, and tangeretin in any sample. There were 85, 139, and 196 SNPs associated with concentrations of flavonoids in peel, pulp, and seed samples, respectively. SNPs associated with concentrations of umbelliferone were only identified in peel samples. Hesperidin, kaempferol, and neoeriocitrin associated SNPs were identified from respective flavonoid concentrations only in seed samples.

**Figure 3:**
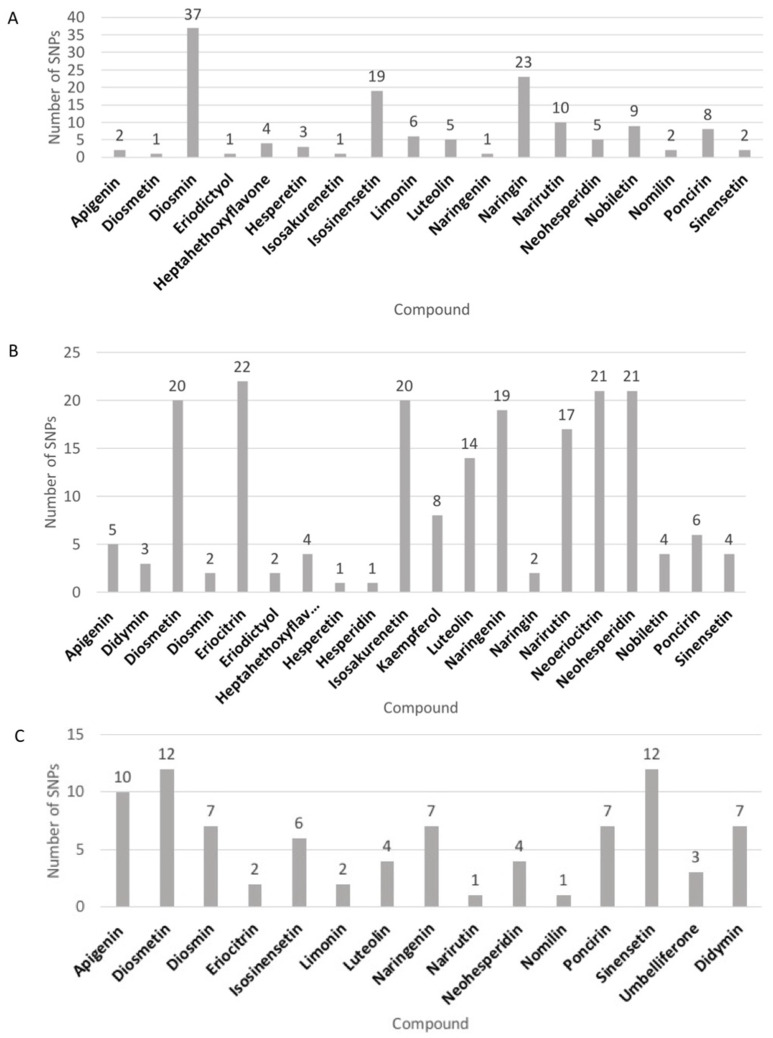
Numbers of significant SNPs identified for each compound in (**A**) pulp sample (**B**) seed sample (**C**) peel sample.

Distribution of compounds and numbers of significant SNPs can be visualized by sample type (Figure 3). In pulp, diosmin had the most and diosmetin, eriodictyol, isosakurenetin, and naringenin had the least number of associated SNPs (Figure 3A). Eriocitrin in seed sample had the highest number, however diosmetin, isoakurenetin, naringenin, narirutin, neoeriocitrin, and neohesperidin had similar numbers of associated SNPs (Figure 3B). Hesperetin and hesperidin had the lowest number of significant SNPs in seed sample (Figure 3B). Diosmetin and sinensetin had the greatest and, narirutin and nomilin had the least, number of associated SNPs in peel sample (Figure 3C).

A summary of the most significant SNPs per compound in each sample also shows a distribution over 9 chromosomes (Table 2). The most significant SNP, associated with kaempferol in seed sample, had a *p*-value of 4.51 × 10^−21^ and was located on chromosome 4 (Table 2). The greatest number of compounds had their most significant SNP located on chromosome 6 and included apignein, didymin, diosmetin, eriodictyol, hesperetin, limonin, luteolin, naringenin, naringin, nomilin, and poncirin (Table 2). Chromosome 3 had nine of the most associated SNPs found for the compounds apigenin, eriocitrin, isosakurenetin, isosinensetin, naringin, neoeriocitrin, nomilin, and sinensetin. Eight compounds (diosmetin, hesperetin, isosinensetin, naringenin, narirutin, neohesperidin, nobiletin, and poncirin) had SNPs with the highest significance associated on chromosome 5 (Table 2). Five of the most significant SNPs were found on chromosome 2 and were associated with diosmin, isosakurenetin, luteolin, narirutin, and nobiletin (Table 2). The chromosomes that had the most significant SNPs associated with four compounds were chromosome 1 (apigenin, diosmetin, diosmin, heptamethoxyflavone) and chromosome 4 (heptamethoxyflavone, kaempferol, limonin, neohesperidin) (Table 2). The three compounds neohesperidin, poncirin, and sinensetin were most associated with SNPs on chromosome 9. Chromosome 7 and 8 each had one significant SNP associated with eriodictyol and diosmin respectively (Table 2). The compounds luteolin, naringenin, and sinensetin had the most significantly associated SNPs, AX-160742943, AX-160808091, and AX-160947519 respectively found in peel and pulp samples (Table 2). SNPs associated with didymin in peel and seed samples had the same SNP (AX-160548920) and highest *p*-value (Table 2).

### 3.3. Candidate Genes

Four candidate genes were selected based on their involvement in flavonoids regulation and biosynthesis in maize, rice, and arabidopsis (Table 3). Candidate genes located on chromosomes 1 and 2, using the Clementine mandarin genome v1.0 as a reference, were significantly associated with heptamethoxyflavone and naringenin in seed samples. The candidate genes found on chromosomes 6 and 3 were associated with naringenin and didymin in peel samples. Gene locations, chromosome number, annotation description, pathway affiliation, Arabidopsis gene ID, predicted citrus ID, and references for flavonoid affiliation are listed in Table 3.

## 4. Discussion

Genome-wide association is a powerful tool that allows plant breeders to dissect complex quantitative traits and provides higher genetic resolution than bi-parental quantitative trait loci (QTL) mapping studies [33]. Citrus breeding is a long-term endeavor, and GWAS provides the potential for genetic marker development based on genome locations associated with difficult to measure traits, that could in turn, accelerate citrus breeding progress. Difficult to measure traits have been the target of GWAS studies in many crops [34,35,36]. Most noteworthy is the use of GWAS for complex nutritional traits, such as flavonoid contents; for example, GWAS was used to identify genes or QTLs associated with total flavonoid content in barley [37], sorghum [38], and rice [39]. This current study is unique in that individual flavonoids of citrus were phenotyped in each sample using a targeted LC-MS/MS approach for their identification and quantification. To our knowledge this is the first study to use a targeted flavonoid (measuring individual compounds) and GWAS approach to uncover associations.

The variation of the flavonoid concentrations that was measured was considerable, with wide ranges from below the limit of detection to over 20,000 µm/g for dry weight sample. Similar ranges were found for targeted flavonoids in juice and edible parts of citrus fruits [40]. For some compounds, the concentrations were not below the detection limit (e.g., tangeretin), however for those compounds no or a few significant SNPs were found (Table 1 and Table 2). This may be due to inadequate variation of the compound in the population, a similar issue is discussed by Caballero et al. [41]. In total we identified 420 significant SNPs associated with 28 flavonoids in peel, pulp, and seed samples. A range in numbers of significant SNPs was found for flavonoids in each sample (Table 2). A study in corn also found variation in significant SNP numbers for two nutritional traits, zinc and iron content [42].

In mandarin pulp samples there were 139 GWAS-identified SNPs associated with 17 compounds (apigening, diosmetin, diosmin, eriodictyol, heptamethoxyflavone, hesperetin, hesperidin, isosakurenetin, isosinensetin, limonin, naringenin, naringin, neohesperidin, nobiletin, nomilin, poncirin, and sinensetin). Mandarin pulp is the part of the fruit that is usually consumed and many compounds with significant SNP associations have been shown to have human health benefits [43]. One of these compounds, nobiletin, had nine significantly associated SNPs and was found at high levels in pulp of some but not all accessions. Nobiletin is a polymethoxylated flavone, which is a class of flavonoids with high absorption and bioactivity [44]. Nobiletin has been extensively studied and has been found to have bioactivities that include inhibiting tumor cell growth, antioxidant activity, inhibition of inflammation, and prevention of cardiovascular diseases. The other flavonoid compounds significantly associated with SNPs have bioactivities similar to nobiletin in pulp [45]. The prevalence of nobiletin in mandarin pulp samples, and its high human absorption, makes it an excellent marker development candidate for mandarin phytonutrient improvement.

Mandarin peel and seed samples had 85 and 196 SNPs significantly associated with targeted flavonoid concentration, respectively. Peel sample had the highest amounts and greatest diversity of compounds of all the sample types tested. Mandarin peel is edible and sometimes used in teas, as an ingredient in some dishes, or as an ingredient in traditional Asian herbal medicines, however, it is not typically consumed. Breeding mandarin varieties with healthful flavonoid compounds in peel and seed may be of use to processors that are interested in high levels of a particular compound to use for processed products (e.g., supplements or powders). In arabidopsis, flavonoid compounds have been shown to elicit plant defense responses against plant pathogens and diseases [46]. In citrus high flavonoid accumulation of tangeretin and naringin inhibited the growth of the fungus *Penicillium digitatum* on citrus peels [47]. Thus, it is possible that marker development and breeding for increased flavonoid content in peel sample may contribute to resistance for fungal diseases such as citrus black spot (*Guignardia citricarpa*), a new problem for fresh citrus growers. It is also important to mention that the significant SNPs found in peel, pulp, and seed sample need to be tested in a segregating population to confirm associations found in this study for flavonoids compounds.

Four candidate genes were found to be involved in flavonoid biosynthesis. The first of these, MYB96, codes for a transcription factor in arabidopsis (Table 3). The MYB96 transcription factor was found to regulate stress responses and flavonoid biosynthesis in a rice model [27]. Recently in citrus, MYB family members were found to regulate flavonoid production [48]. The predicted MYB96 location in the Clementine genome is on chromosome 3 at LOC18048626 (Table 3). There were other SNPs that mapped to MYB transcription factors, however enrichment analysis showed no association between other MYB genes and flavonoid biosynthesis. The two genes, MPL12.14 and AT3G29635, were also found to be involved in flavonoid biosynthesis from the enrichment analysis. However, there were no studies in the literature that directly related MPL12.14 and AT3G29635 to flavonoid accumulation. The gene MPL12.14 was found in arabidopsis to be involved with the synthesis of phenylpropanoids for the construction of cell wall components [28]. The gene AT3G29635 was found to be involved with anthocyanin acyltransferases and phenylpropanoid regulation in Arabidopsis [30]. It may be that MPL12.14 and AT3G29635 are predicted to be involved in regulation of flavonoids, but there have not been any studies to validate their role in citrus. The homologous genes for MPL12.14 and AT3G29635 in *Citrus clementina* are on chromosomes 2 and 1 at locations LOC18053255 and LOC18055186 respectively. The fourth candidate gene found was CRTISO which was mapped to LOC18038313 in the *Citrus clementina* genome and regulates carotenoid synthesis. In addition, CRTISO was found to be involved in flavonoid biosynthesis and regulation in arabidopsis and maize [31,32].

In other citrus flavonoid studies, genes such as chalcone synthase (CHS) and chalcone isomerase, that are upstream in the biosynthetic pathway of a given flavonoid, have been identified and shown to regulate flavonoid biosynthesis [49,50]. Citrus contains many unique flavonoids and genetic regulators of downstream products of the flavonoid pathways that have not been identified because many of them do not exist in model plants. More studies with citrus mutants are needed to elucidate genes in the flavonoid pathway that are responsible for the synthesis of unique citrus flavonoids. Flavonoids not only play an important role in optimizing human health but are also linked to plant disease and pest tolerance. Recently Killiny et al. [51] found that citrus cultivars with greater levels of volatile and non-volatile compounds may have greater tolerance to Huanglongbing (HLB). The relationship between HLB tolerance and flavonoid contents have yet to be elucidated. However, there are studies that show there are higher levels of flavonoids in fruits from HLB-infected trees [52]. Flavonoid association studies can play an important role in breeding for superior plant and human health. Genetic components have an important role in the regulation of citrus flavonoids, and this foundational study can be used to further validate and investigate regulation of the citrus flavonoid pathways.

## Figures and Tables

**Table 1:** Concentration range of 28 flavonoid compounds detected in peel, pulp, and seed samples from diverse mandarin accessions.

#	Compound	Peel	Peel	Peel	Pulp	Pulp	Pulp	Seed	Seed	Seed
		Mean	Max.	Min.	Mean	Max.	Min.	Mean	Max.	Min.
1	Apigenin	0.01 ^z^	1.88	0	0.0002	0.03	0	0.0004	0.07	0
2	Coumarin	0.006	0.1	0	0.0007	0.16	0	0	0	0
3	Didymin	3.71	118.59	0	5.19	133.51	0.005	0.12	2.93	0
4	Diosmetin	0.56	58.99	0	0.008	0.88	0	0.008	0.35	0
5	Diosmin	0.003	0.02	0	0.0006	0.06	0	0.02	0.46	0
6	Eriocitrin	0.32	9.16	0	0.35	22.55	0	0.03	2.03	0
7	Eriodictyol	0.007	0.04	0	0.05	0.05	0	0.0001	0.01	0
8	Heptamethoxyflavone	50.7	1767.33	0	3.17	148.92	0.03	2.37	54.59	0.16
9	Hesperetin	0.05	11.28	0	0.003	0.18	0	0.02	0.94	0
10	Hesperidin	14.5	575.78	0.05	14.07	266.89	0.02	0.55	17.3	0.002
11	Isosakurenetin	0.005	0.07	0	0.0004	0.1	0	0.0005	0.05	0
12	Isosinensetin	82.99	1372.64	0	3.47	76.36	0	0.32	10.95	0
13	Kaempferol	0	0	0	0	0	0	0.0006	0.08	0
14	Limonin	9.2	231.94	0	13.65	410.42	0.03	148.17	718.93	5.24
15	Luteolin	0.002	0.04	0	0.0006	0.07	0	0.001	0.04	0
16	Naringenin	0.03	1.43	0	0.004	0.45	0	0.0006	0.07	0
17	Naringin	0.25	14.05	0	0.4	20.77	0	0.02	2.35	0
18	Narirutin	4.91	125.13	0	6.69	243.81	0	0.31	6.21	0
19	Neodiosmin	12.41	321.22	0	4.22	108.55	0	0.54	3.46	0.03
20	Neoeriocitrin	0.01	1.49	0	0.02	1.84	0	0.01	0.86	0
21	Neohesperidin	1.95	58.19	0	2.59	86.45	0	0.08	4.67	0
22	Nobiletin	471.84	0.02	0.03	28.72	597.4	0.32	18.73	204.64	2.08
23	Nomilin	0.88	39.72	0	1.86	43.07	0	24.56	134.1	0.24
24	Poncirin	0.16	14.65	0	0.19	11.32	0	0.11	20.42	0
25	Rutin	0.16	3.72	0	0.04	0.62	0	0.05	0.38	0
26	Sinensetin	42.43	1302.52	0	2.1	83.56	0	0.167	13.62	0
27	Tangeretin	444.28	0.98	4.86	152.36	1989.3	0.38	175.13	1218.65	10.36
28	Umbelliferone	0.006	0.13	0	0	0	0	0	0	0

^z^ Concentrations are expressed in µg/g of dry weight.

**Table 2:** The most significant GWAS-identified SNPs found for each compound in each sample type.

Compound	Sample Type	Marker	Chromosome	Position	Gene	Proportion of Variance Explained	*p*-Value
Apigenin	peel	AX-160026423	1	28455844	Ciclev10007429m.g	0.065	2.72 × 10^−7^
Apigenin	pulp	AX-161017449	3	8373806	Ciclev10019809m.g	0.066	1.41 × 10^−7^
Apigenin	seed	AX-160120038	6	18733554	Ciclev10013344m.g	0.070	1.57 × 10^−7^
Didymin	seed	AX-160548920	6	500457	Ciclev10011912m.g	0.080	1.69 × 10^−8^
Didymin	peel	AX-160548920	6	500457	Ciclev10011912m.g	0.070	4.94 × 10^−8^
Diosmetin	seed	AX-160428826	5	34679192	Ciclev10003655m.g	0.195	9.47 × 10^−20^
Diosmetin	peel	AX-159831918	1	27881393	Ciclev10009180m.g	0.048	1.13 × 10^−8^
Diosmetin	pulp	AX-160808091	6	23828780	Ciclev10011357m.g	0.052	2.99 × 10^−6^
Diosmin	pulp	AX-160953115	1	4957396	Ciclev10007412m.g	0.103	2.98 × 10^−11^
Diosmin	peel	AX-160796087	8	23855395	Ciclev10029047m.g	0.059	5.87 × 10^−7^
Diosmin	seed	AX-160362613	2	35355786	Ciclev10014319m.g	0.060	1.13 × 10^−6^
Eriocitrin	seed	AX-160981611	3	5235434	Ciclev10021722m.g	0.095	7.55 × 10^−10^
Eriocitrin	peel	AX-160487850	3	35789790	Ciclev10020864m.g	0.068	8.05 × 10^−8^
Eriodictyol	seed	AX-160014487	7	1494553	Ciclev10025311m.g	0.094	8.81 × 10^−10^
Eriodictyol	pulp	AX-160808091	6	23828780	Ciclev10011357m.g	0.052	2.99 × 10^−6^
Heptamethoxyflavone	pulp	AX-160478010	4	23838038	Ciclev10032432m.g	0.073	2.9 × 10^−8^
Heptamethoxyflavone	seed	AX-159875671	1	23588919	Ciclev10008059m.g	0.067	2.91 × 10^−7^
Hesperetin	pulp	AX-159872199	6	24383951	Ciclev10012224m.g	0.059	5.63 × 10^−7^
Hesperetin	seed	AX-160906983	5	13059166	Ciclev10002443m.g	0.058	2.05 × 10^−6^
Hesperidin	seed	AX-160261315	5	34690939	Ciclev10002520m.g	0.070	1.39 × 10^−7^
Hesperidin	Pulp	AX-159823569	7	92972	Ciclev10025023m.g	0.050	4.85 × 10^−6^
Isosakurenetin	seed	AX-159926254	2	12136093	Ciclev10014778m.g	0.066	3.27 × 10^−7^
Isosakurenetin	pulp	AX-160865092	3	40172143	Ciclev10024487m.g	0.054	1.96 × 10^−6^
Isosinensetin	pulp	AX-160385265	5	34255060	Ciclev10001494m.g	0.077	1.82 × 10^−8^
Isosinensetin	peel	AX-160729031	3	27830592	Ciclev10019217m.g	0.053	2.39 × 10^−6^
Kaempferol	seed	AX-159946295	4	18414143	Ciclev10032158m.g	0.208	4.51 × 10^−21^
Limonin	peel	AX-160548920	6	500457	Ciclev10011912m.g	0.076	1.24 × 10^−8^
Limonin	pulp	AX-160121385	4	21318491	Ciclev10033394m.g	0.054	8.40 × 10^−6^
Luteolin	seed	AX-159825934	2	3781683	Ciclev10016037m.g	0.294	1.07 × 10^−30^
Luteolin	pulp	AX-160742943	6	20232219	Ciclev10012009m.g	0.067	1.11 × 10^−7^
Luteolin	peel	AX-160742943	6	20232219	Ciclev10012009m.g	0.065	1.73 × 10^−7^
Naringenin	peel	AX-160808091	6	23828780	Ciclev10011357m.g	0.070	7.57 × 10^−8^
Naringenin	seed	AX-159951409	5	24061380	Ciclev10001582m.g	0.068	2.38 × 10^−7^
Naringenin	pulp	AX-160808091	6	23828780	Ciclev10011357m.g	0.063	2.49 × 10^−7^
Naringin	pulp	AX-160259155	3	42212133	Ciclev10022115m.g	0.127	1.02 × 10^−13^
Naringin	seed	AX-159969067	6	25181051	Ciclev10011245m.g	0.060	1.30 × 10^−6^
Narirutin	seed	AX-160529374	5	24023209	Ciclev10001022m.g	0.099	3.28 × 10^−10^
Narirutin	pulp	AX-160871858	2	10250942	Ciclev10016536m.g	0.057	1.79 × 10^−6^
Neoeriocitrin	seed	AX-160981611	3	5235434	Ciclev10021722m.g	0.090	1.89 × 10^−9^
Neohesperidin	pulp	AX-160292039	4	7374156	Ciclev10033003m.g	0.119	6.81 × 10^−13^
Neohesperidin	peel	AX-159868580	5	21083453	Ciclev10001079m.g	0.100	5.47 × 10^−11^
Neohesperidin	seed	AX-160395823	9	21603308	Ciclev10005135m.g	0.090	2.29 × 10^−9^
Nobiletin	pulp	AX-160014948	5	33018623	Ciclev10000065m.g	0.060	5.61 × 10^−7^
Nobiletin	seed	AX-160432194	2	27643973	Ciclev10014906m.g	0.058	2.27 × 10^−6^
Nomilin	peel	AX-160423747	6	17478449	Ciclev10011307m.g	0.066	1.19 × 10^−7^
Nomilin	pulp	AX-160298860	3	3171806	Ciclev10022532m.g	0.060	5.22 × 10^−7^
Poncirin	seed	AX-159969067	6	25181051	Ciclev10011245m.g	0.098	3.39 × 10^−10^
Poncirin	peel	AX-160261315	5	34690939	Ciclev10002520m.g	0.063	2.68 × 10^−7^
Poncirin	pulp	AX-160395823	9	21603308	Ciclev10005135m.g	0.060	4.68 × 10^−7^
Sinensetin	peel	AX-160947519	9	29959571	Ciclev10006155m.g	0.106	1.25 × 10^−11^
Sinensetin	seed	AX-160284313	3	40137702	Ciclev10020460m.g	0.077	3.48 × 10^−8^
Sinensetin	pulp	AX-160947519	9	29959571	Ciclev10006155m.g	0.070	5.58 × 10^−8^

**Table 3:** GWAS identified SNPs that were found with enrichment analysis.

Associated Compound/Samples	“Given Name”	GWAS Identified SNP	Chr.	Physical Position	Annotation	Arabidopsis Orthologue	Citrus Gene	Citrus LOC	Predicted Citrus Annotation	Study References
Didymin/peel	MYB96	AX-160475114	3	7201172	Myb transcription factor	AT5G62470	Ciclev10020967m.g	LOC18048626	Citrus clementina myb-related protein 306	[27]
Naringenin/seed	MPL12.14	AX-161026143	2	34869109	protein REDUCED WALL ACETYLATION 1	AT5G46340	Ciclev10014824m.g	LOC18053255	REDUCED WALL ACETYLATION 3	[28,29]
Heptamethoxyflavone/seed	AT3G29635	AX-159875671	1	23596472	HXXXD-type acyl-transferase-like protein	AT3G29635	Ciclev10008059m.g	LOC18055186	malonyl-CoA:anthocyanidin 5-O-glucoside-6″-O-malonyltransferase	[30]
Naringenin/peel	CRTISO	AX-160782343	6	19581218	carotenoid isomerase	AT1G06820	Ciclev10011230m.g	LOC18038313	prolycopene isomerase, chloroplastic	[31,32]

## Data Availability

Due to confidentiality agreements, supporting data can only be made available to researchers subject to a non-disclosure agreement. Details of the data and how to request access are available from fgmitter@ufl.edu at University of Florida.

## Supplementary Materials

The following supporting information can be downloaded at: https://www.mdpi.com/article/10.3390/plants11030317/s1, Figure S1: Genome Wide Association Manhattan and Quantile-Quantile Plots Presented by Compound for Significant SNPS; Table S1: Diverse mandarin accessions used for the genome wide association of healthful flavonoids; Table S2: List of GWAS determined SNPs in mandarin peel, pulp, and seed.
