# Supplementary material for: Genome-Wide Association Study of Healthful Flavonoids among Diverse Mandarin Accessions
Source: Plants (Basel). 2022 Jan 25;11(3):317. doi: 10.3390/plants11030317 (PMC8839032; doi:10.3390/plants11030317)
Supplement: Supplementary file 1 [file plants-11-00317-s001.zip › GWAS Figure S1.pdf]

Figure S1. Genome Wide Association Manhattan and Quantile-Quantile Plots Presented by Compound for Significant SNPS

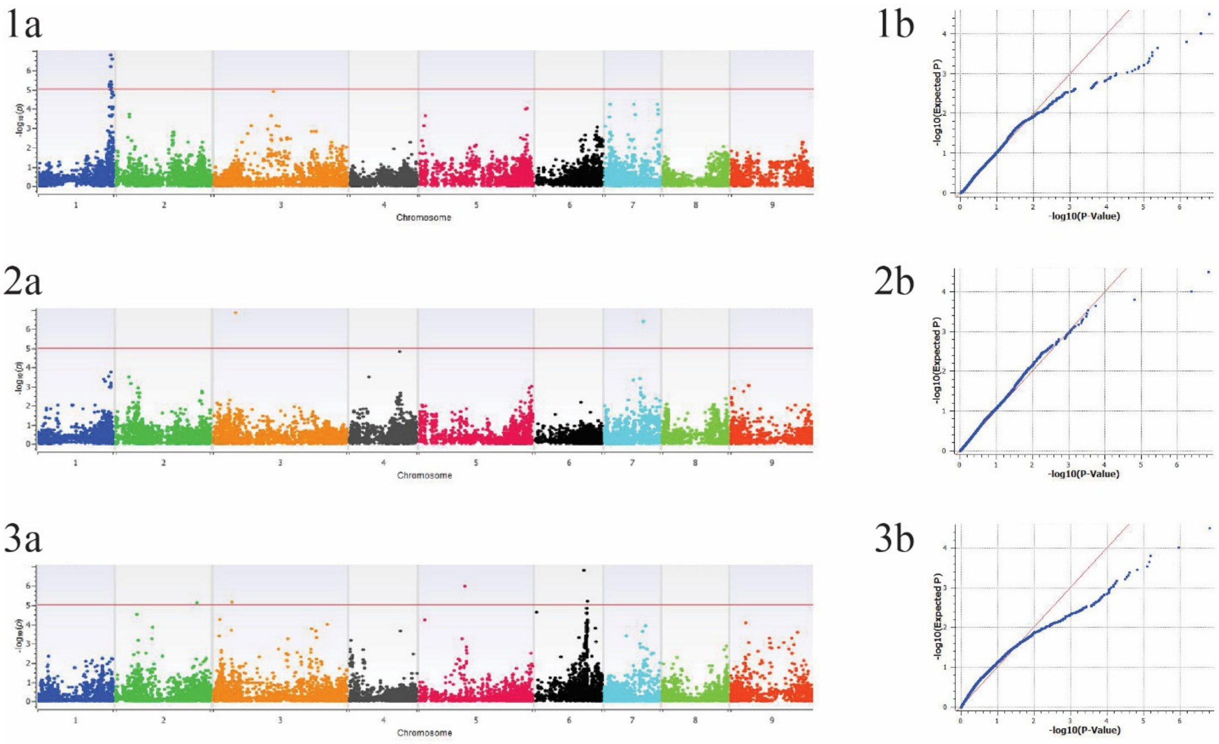

Figure S1-1. Genome wide association analysis of apigenin (1a) Manhattan plot for GWAS in mandarin peel tissue on chromosomes 1-9 (1b) Quantile-quantile plot for the GWAS in mandarin peel tissues under MLM. The horizontal axis shows –log10 transformed observed *P* value, while the vertical axis indicates –log10 transformed expected *P* value. (2a) Manhattan plot for GWAS in mandarin pulp tissue on chromosomes 1-9 (2b) Quantile-quantile plot for the GWAS in mandarin pulp tissues under MLM. The horizontal axis shows –log10 transformed observed P value, while the vertical axis indicates –log10 transformed expected P value. (3a) Manhattan plot for GWAS in mandarin seed tissue on chromosomes 19-9 (3b) Quantile-quantile plot for the GWAS in mandarin seed tissues under MLM. The horizontal axis shows –log10 transformed observed P value, while the vertical axis indicates –log10 transformed expected P value. The horizontal red line in Manhattan plots indicates a threshold value for significant SNPs. All SNPs with –log10 (P values) greater than 4.9 were considered significant.

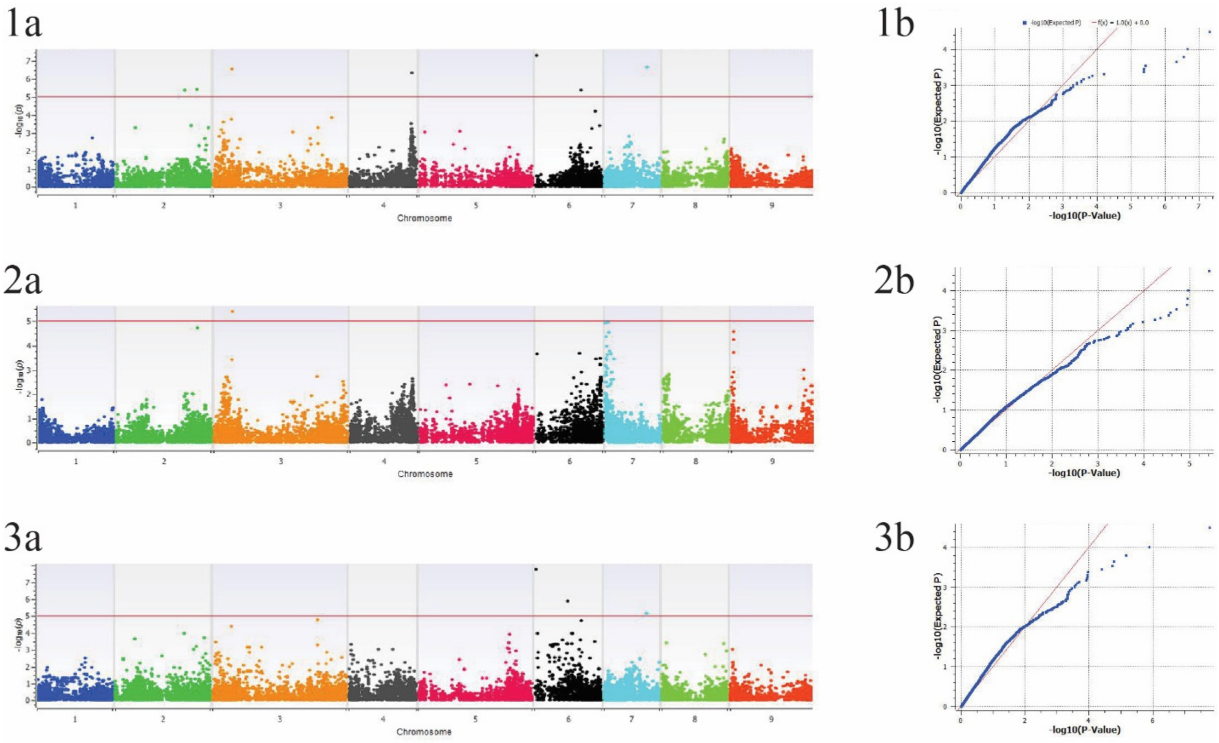

Figure S1-2. Genome wide association analysis of didymin (1a) Manhattan plot for GWAS in mandarin peel tissue on chromosomes 1-9 (1b) Quantile-quantile plot for the GWAS in mandarin peel tissues under MLM. The horizontal axis shows –log10 transformed observed *P* value, while the vertical axis indicates –log10 transformed expected *P* value. (2a) Manhattan plot for GWAS in mandarin pulp tissue on chromosomes 1-9 (2b) Quantile-quantile plot for the GWAS in mandarin pulp tissues under MLM. The horizontal axis shows –log10 transformed observed P value, while the vertical axis indicates –log10 transformed expected P value. (3a) Manhattan plot for GWAS in mandarin seed tissue on chromosomes 1-9 (3b) Quantile-quantile plot for the GWAS in mandarin seed tissues under MLM. The horizontal axis shows –log10 transformed observed P value, while the vertical axis indicates –log10 transformed expected P value. The horizontal red line in Manhattan plots indicates a threshold value for significant SNPs. All SNPs with –log10 (P values) greater than 4.9 were considered significant.

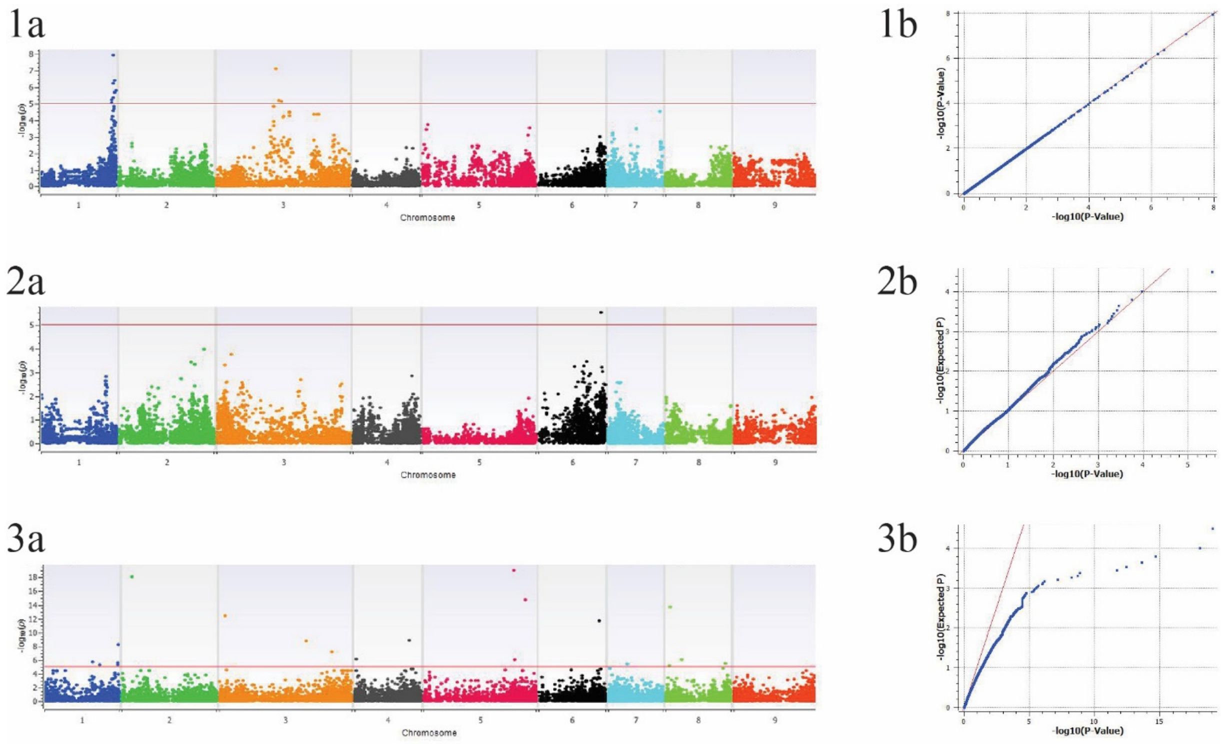

Figure S1-3. Genome wide association analysis of diosmetin (1a) Manhattan plot for GWAS in mandarin peel tissue on chromosomes 1-9 (1b) Quantile-quantile plot for the GWAS in mandarin peel tissues under MLM. The horizontal axis shows –log10 transformed observed *P* value, while the vertical axis indicates –log10 transformed expected *P* value. (2a) Manhattan plot for GWAS in mandarin pulp tissue on chromosomes 1-9 (2b) Quantile-quantile plot for the GWAS in mandarin pulp tissues under MLM. The horizontal axis shows –log10 transformed observed P value, while the vertical axis indicates –log10 transformed expected P value. (3a) Manhattan plot for GWAS in mandarin seed tissue on chromosomes 1-9 (3b) Quantile-quantile plot for the GWAS in mandarin seed tissues under MLM. The horizontal axis shows –log10 transformed observed P value, while the vertical axis indicates –log10 transformed expected P value. The horizontal red line in Manhattan plots indicates a threshold value for significant SNPs. All SNPs with –log10 (P values) greater than 4.9 were considered significant.

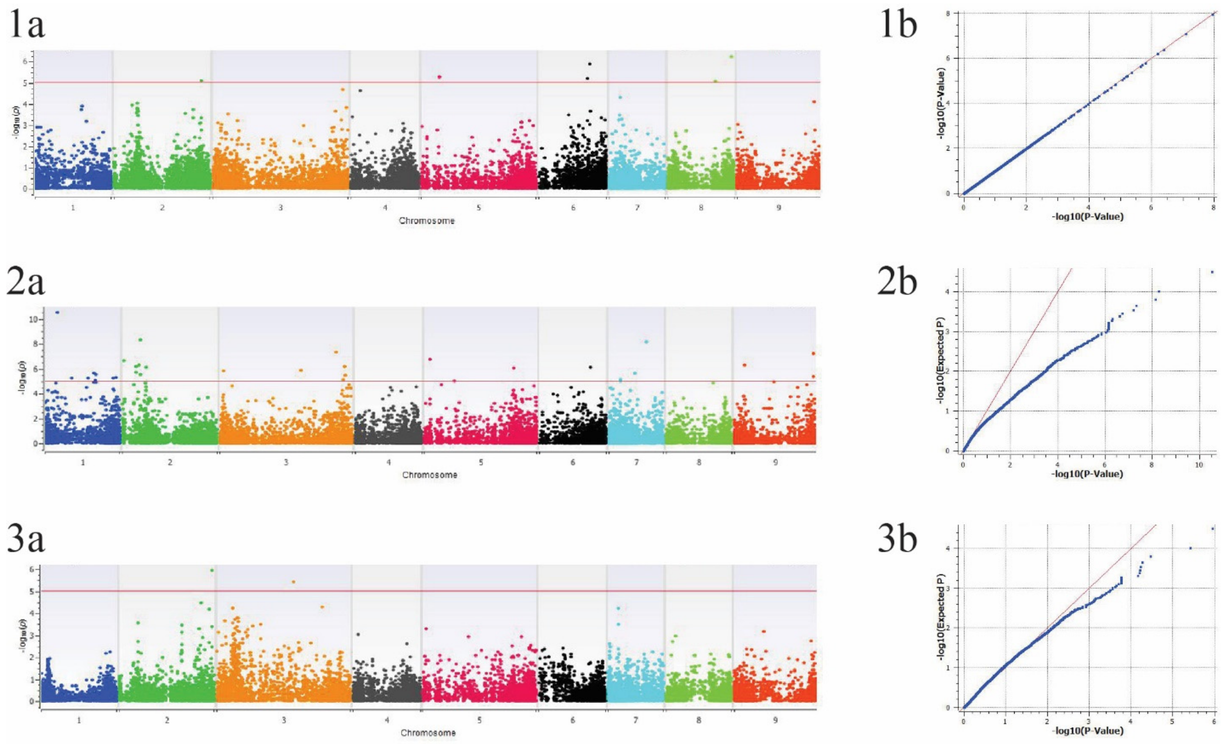

Figure S1-4. Genome wide association analysis of diosmin (1a) Manhattan plot for GWAS in mandarin peel tissue on chromosomes 1-9 (1b) Quantile-quantile plot for the GWAS in mandarin peel tissues under MLM. The horizontal axis shows –log10 transformed observed *P* value, while the vertical axis indicates –log10 transformed expected *P* value. (2a) Manhattan plot for GWAS in mandarin pulp tissue on chromosomes 1-9 (2b) Quantile-quantile plot for the GWAS in mandarin pulp tissues under MLM. The horizontal axis shows –log10 transformed observed P value, while the vertical axis indicates –log10 transformed expected P value. (3a) Manhattan plot for GWAS in mandarin seed tissue on chromosomes 1-9 (3b) Quantile-quantile plot for the GWAS in mandarin seed tissues under MLM. The horizontal axis shows –log10 transformed observed P value, while the vertical axis indicates –log10 transformed expected P value. The horizontal red line in Manhattan plots indicates a threshold value for significant SNPs. All SNPs with –log10 (P values) greater than 4.9 were considered significant.

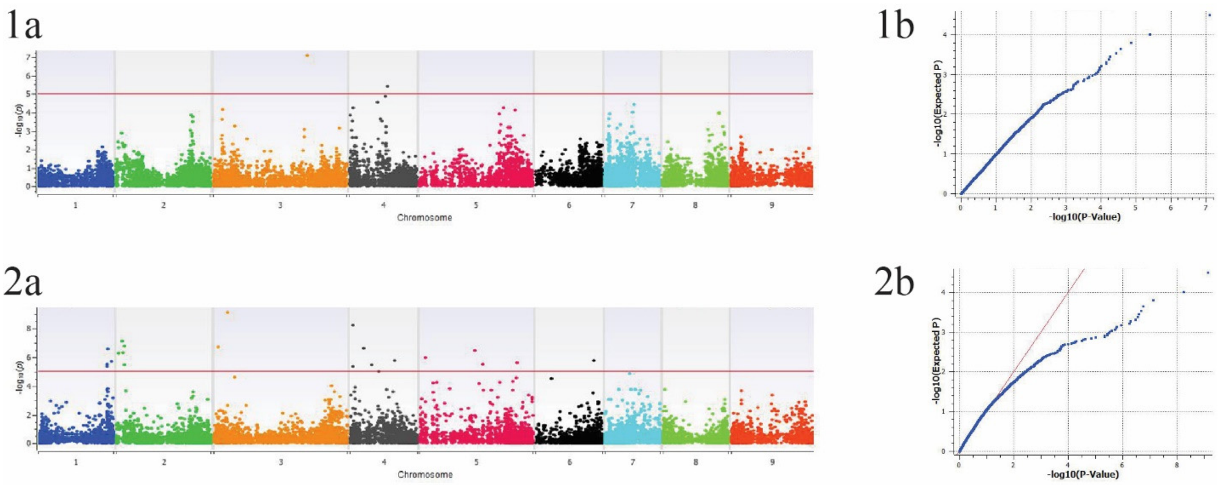

Figure S1-5. Genome wide association analysis of eriocitrin (1a) Manhattan plot for GWAS in mandarin peel tissue on chromosomes 1-9 (1b) Quantile-quantile plot for the GWAS in mandarin peel tissues under MLM. The horizontal axis shows –log10 transformed observed P value, while the vertical axis indicates –log10 transformed expected P value. (2a) Manhattan plot for GWAS in mandarin seed tissue on chromosomes 1-9 (2b) Quantile-quantile plot for the GWAS in mandarin seed tissues under MLM. The horizontal axis shows –log10 transformed observed P value, while the vertical axis indicates –log10 transformed expected P value. The horizontal red line in Manhattan plots indicates a threshold value for significant SNPs. All SNPs with –log10 (P values) greater than 4.9 were considered significant.

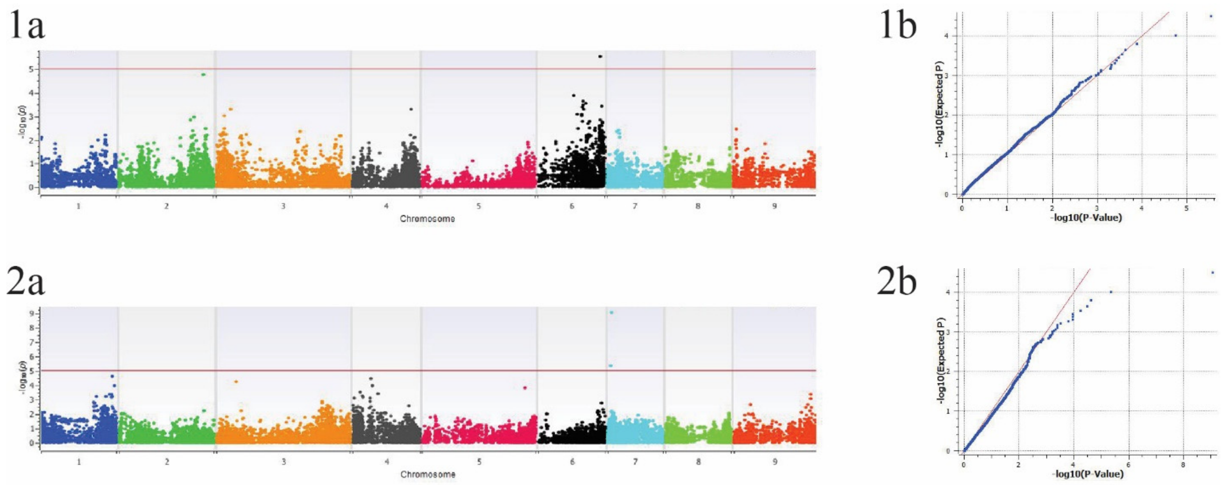

Figure S1-6. Genome wide association analysis of eriodictyol (1a) Manhattan plot for GWAS in mandarin pulp tissue on chromosomes 1-9 (1b) Quantile-quantile plot for the GWAS in mandarin pulp tissues under MLM. The horizontal axis shows –log10 transformed observed P value, while the vertical axis indicates –log10 transformed expected P value. (2a) Manhattan plot for GWAS in mandarin seed tissue on chromosomes 1-9 (2b) Quantile-quantile plot for the GWAS in mandarin seed tissues under MLM. The horizontal axis shows –log10 transformed observed P value, while the vertical axis indicates –log10 transformed expected P value. The horizontal red line in Manhattan plots indicates a threshold value for significant SNPs. All SNPs with –log10 (P values) greater than 4.9 were considered significant.

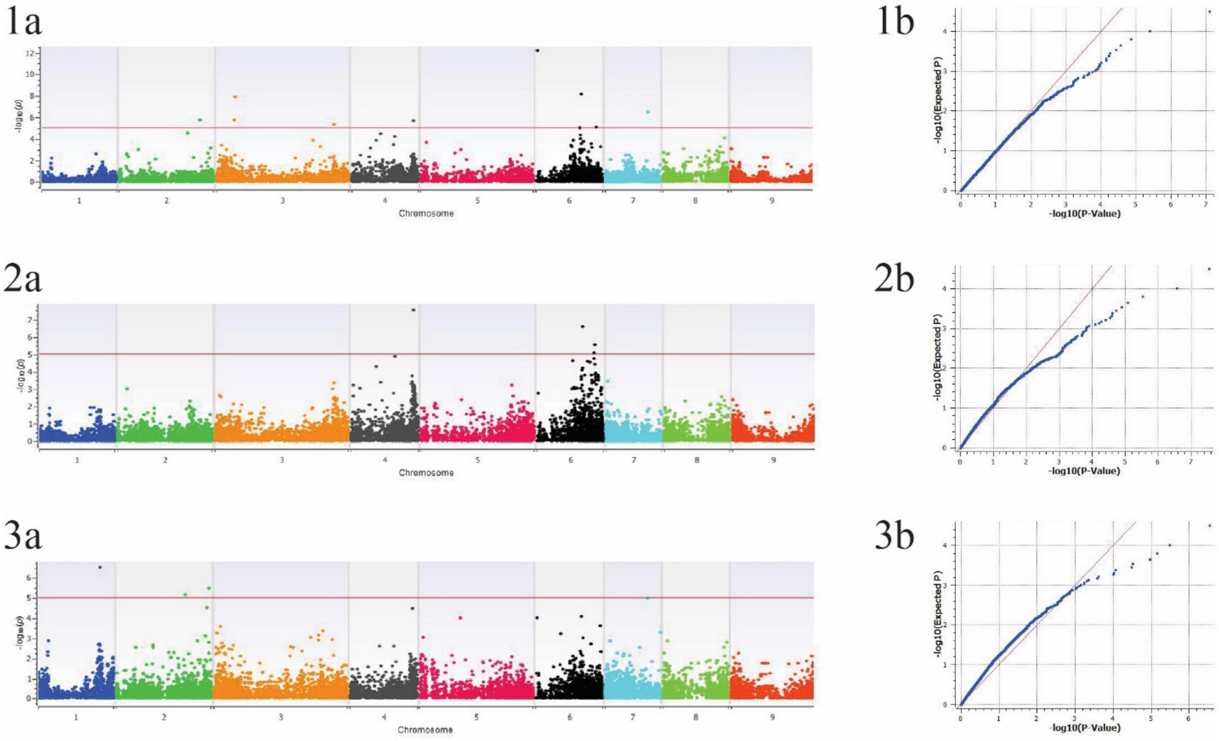

Figure S1-7. Genome wide association analysis of heptamethoxyflavone (1a) Manhattan plot for GWAS in mandarin peel tissue on chromosomes 1-9 (1b) Quantile-quantile plot for the GWAS in mandarin peel tissues under MLM. The horizontal axis shows –log10 transformed observed *P* value, while the vertical axis indicates –log10 transformed expected *P* value. (2a) Manhattan plot for GWAS in mandarin pulp tissue on chromosomes 1-9 (2b) Quantile-quantile plot for the GWAS in mandarin pulp tissues under MLM. The horizontal axis shows –log10 transformed observed P value, while the vertical axis indicates –log10 transformed expected P value. (3a) Manhattan plot for GWAS in mandarin seed tissue on chromosomes 1-9 (3b) Quantile-quantile plot for the GWAS in mandarin seed tissues under MLM. The horizontal axis shows –log10 transformed observed P value, while the vertical axis indicates –log10 transformed expected P value. The horizontal red line in Manhattan plots indicates a threshold value for significant SNPs. All SNPs with –log10 (P values) greater than 4.9 were considered significant.

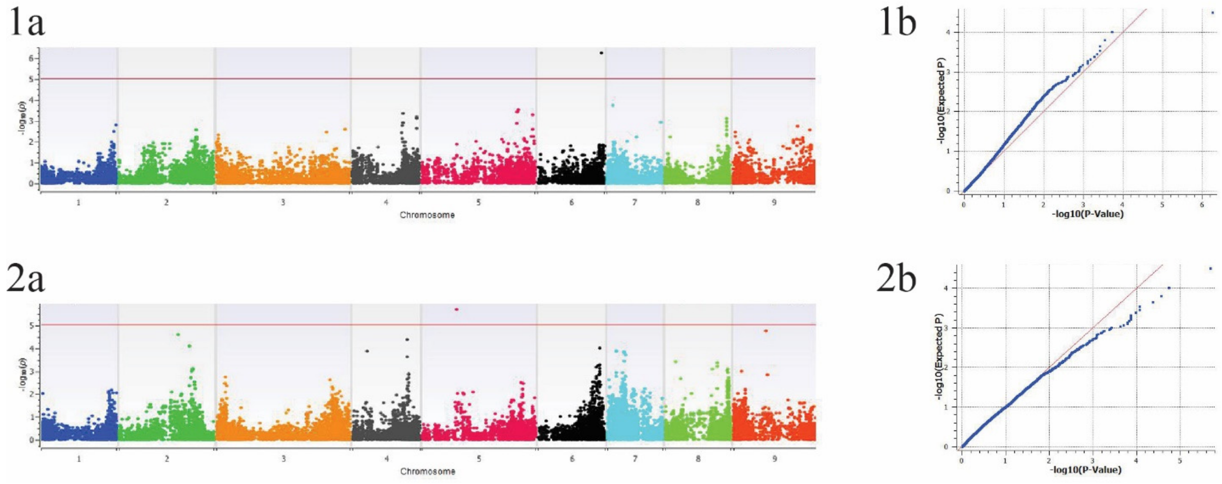

Figure S1-8. Genome wide association analysis of hesperetin (1a) Manhattan plot for GWAS in mandarin pulp tissue on chromosomes 1-9 (1b) Quantile-quantile plot for the GWAS in mandarin pulp tissues under MLM. The horizontal axis shows –log10 transformed observed P value, while the vertical axis indicates –log10 transformed expected P value. (2a) Manhattan plot for GWAS in mandarin seed tissue on chromosomes 1-9 (2b) Quantile-quantile plot for the GWAS in mandarin seed tissues under MLM. The horizontal axis shows –log10 transformed observed P value, while the vertical axis indicates –log10 transformed expected P value. The horizontal red line in Manhattan plots indicates a threshold value for significant SNPs. All SNPs with –log10 (P values) greater than 4.9 were considered significant.

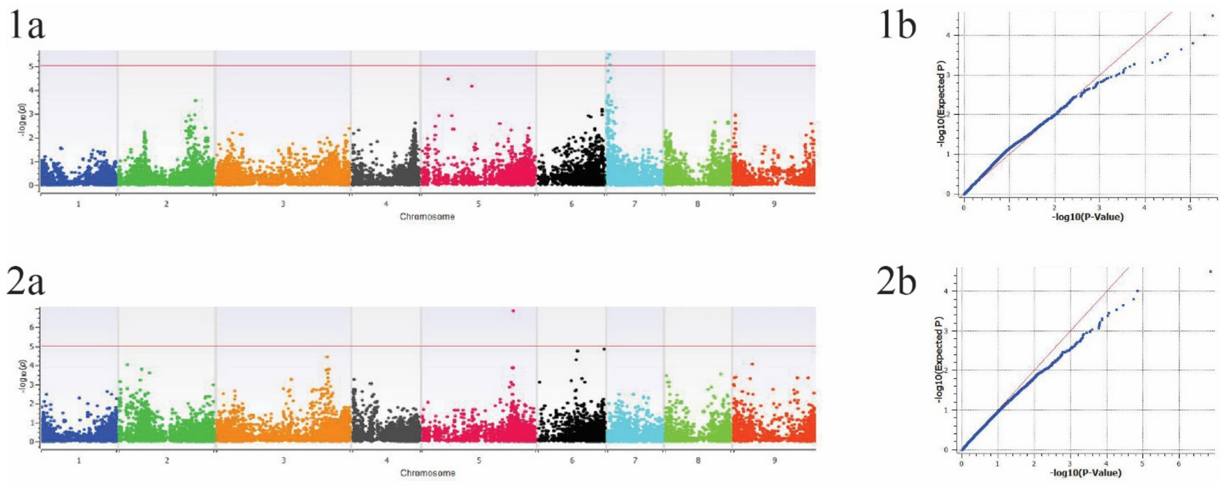

Figure S1-9. Genome wide association analysis of hesperidin (1a) Manhattan plot for GWAS in mandarin pulp tissue on chromosomes 1-9 (1b) Quantile-quantile plot for the GWAS in mandarin pulp tissues under MLM. The horizontal axis shows –log10 transformed observed P value, while the vertical axis indicates –log10 transformed expected P value. (2a) Manhattan plot for GWAS in mandarin seed tissue on chromosomes 1-9 (2b) Quantile-quantile plot for the GWAS in mandarin seed tissues under MLM. The horizontal axis shows –log10 transformed observed P value, while the vertical axis indicates –log10 transformed expected P value. The horizontal red line in Manhattan plots indicates a threshold value for significant SNPs. All SNPs with –log10 (P values) greater than 4.9 were considered significant.

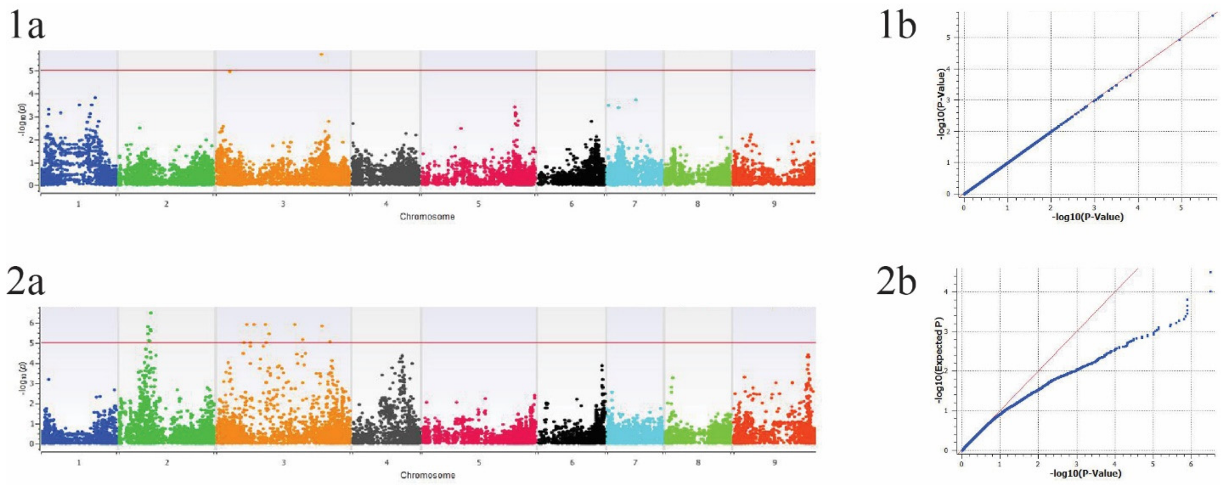

Figure S1-10. Genome wide association analysis of isosakurenetin (1a) Manhattan plot for GWAS in mandarin pulp tissue on chromosomes 1-9 (1b) Quantile-quantile plot for the GWAS in mandarin pulp tissues under MLM. The horizontal axis shows –log10 transformed observed P value, while the vertical axis indicates –log10 transformed expected P value. (2a) Manhattan plot for GWAS in mandarin seed tissue on chromosomes 1-9 (2b) Quantile-quantile plot for the GWAS in mandarin seed tissues under MLM. The horizontal axis shows –log10 transformed observed P value, while the vertical axis indicates –log10 transformed expected P value. The horizontal red line in Manhattan plots indicates a threshold value for significant SNPs. All SNPs with –log10 (P values) greater than 4.9 were considered significant.

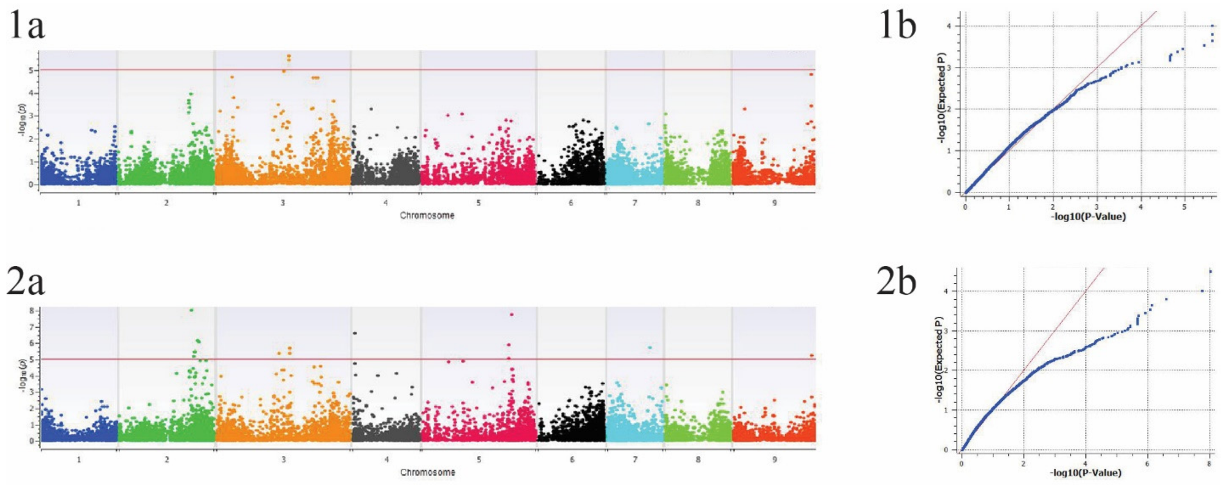

Figure S1-11. Genome wide association analysis of isosinenesetin (1a) Manhattan plot for GWAS in mandarin peel tissue on chromosomes 1-9 (1b) Quantile-quantile plot for the GWAS in mandarin peel tissues under MLM. The horizontal axis shows –log10 transformed observed *P* value, while the vertical axis indicates –log10 transformed expected *P* value. (2a) Manhattan plot for GWAS in mandarin pulp tissue on chromosomes 1-9 (2b) Quantile-quantile plot for the GWAS in mandarin pulp tissues under MLM. The horizontal axis shows –log10 transformed observed P value, while the vertical axis indicates –log10 transformed expected P value. The horizontal red line in Manhattan plots indicates a threshold value for significant SNPs. All SNPs with –log10 (P values) greater than 4.9 were considered significant.

1a

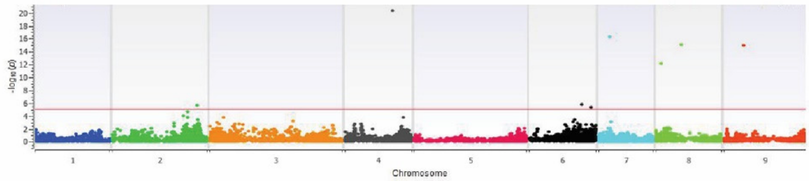

1b

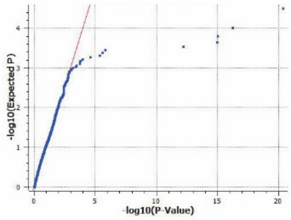

Figure S1-12. Genome wide association analysis of kaempferol (1a) Manhattan plot for GWAS in mandarin seed tissue on chromosomes 1-9 (1b) Quantile-quantile plot for the GWAS in mandarin seed tissues under MLM. The horizontal axis shows –log10 transformed observed P value, while the vertical axis indicates –log10 transformed expected P value. The horizontal red line in Manhattan plots indicates a threshold value for significant SNPs. All SNPs with –log10 (P values) greater than 4.9 were considered significant.

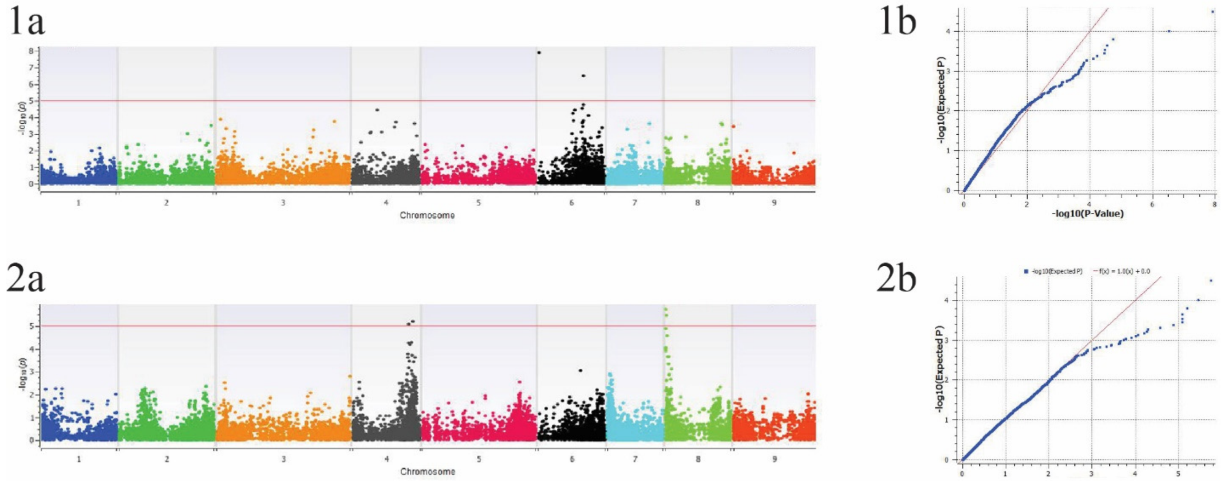

Figure S1-13. Genome wide association analysis of limonin (1a) Manhattan plot for GWAS in mandarin peel tissue on chromosomes 1-9 (1b) Quantile-quantile plot for the GWAS in mandarin peel tissues under MLM. The horizontal axis shows –log10 transformed observed *P* value, while the vertical axis indicates –log10 transformed expected *P* value. (2a) Manhattan plot for GWAS in mandarin pulp tissue on chromosomes 1-9 (2b) Quantile-quantile plot for the GWAS in mandarin pulp tissues under MLM. The horizontal axis shows –log10 transformed observed P value, while the vertical axis indicates –log10 transformed expected P value. The horizontal red line in Manhattan plots indicates a threshold value for significant SNPs. All SNPs with –log10 (P values) greater than 4.9 were considered significant.

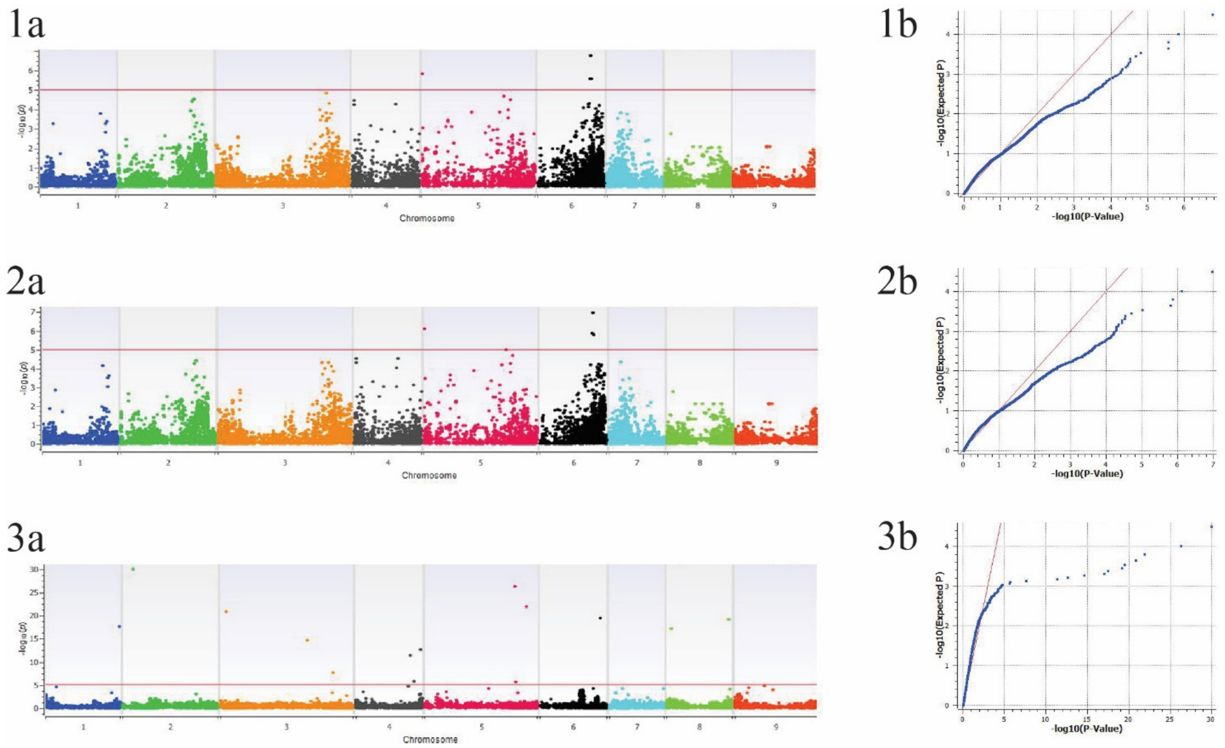

Figure S1-14. Genome wide association analysis of luteolin (1a) Manhattan plot for GWAS in mandarin peel tissue on chromosomes 1-9 (1b) Quantile-quantile plot for the GWAS in mandarin peel tissues under MLM. The horizontal axis shows –log10 transformed observed *P* value, while the vertical axis indicates –log10 transformed expected *P* value. (2a) Manhattan plot for GWAS in mandarin pulp tissue on chromosomes 1-9 (2b) Quantile-quantile plot for the GWAS in mandarin pulp tissues under MLM. The horizontal axis shows –log10 transformed observed P value, while the vertical axis indicates –log10 transformed expected P value. (3a) Manhattan plot for GWAS in mandarin seed tissue on chromosomes 1-9 (3b) Quantile-quantile plot for the GWAS in mandarin seed tissues under MLM. The horizontal axis shows –log10 transformed observed P value, while the vertical axis indicates –log10 transformed expected P value. The horizontal red line in Manhattan plots indicates a threshold value for significant SNPs. All SNPs with –log10 (P values) greater than 4.9 were considered significant.

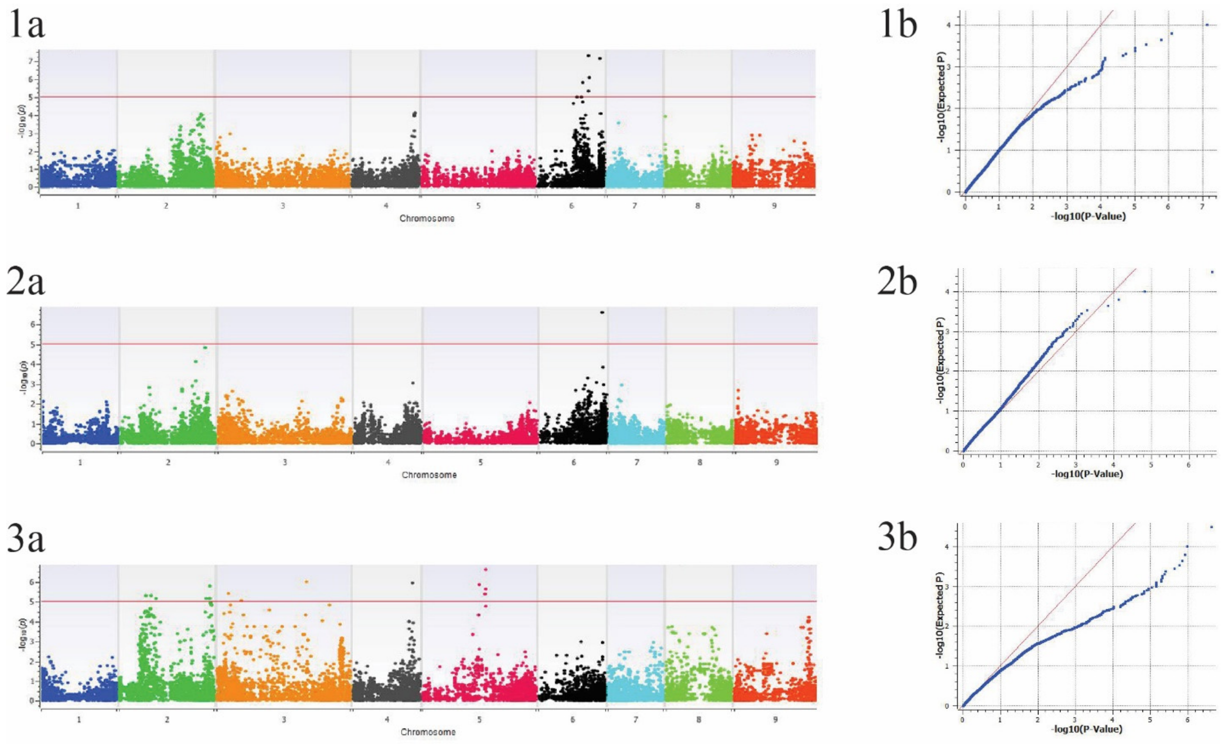

Figure S1-15. Genome wide association analysis of naringenin (1a) Manhattan plot for GWAS in mandarin peel tissue on chromosomes 1-9 (1b) Quantile-quantile plot for the GWAS in mandarin peel tissues under MLM. The horizontal axis shows –log10 transformed observed *P* value, while the vertical axis indicates –log10 transformed expected *P* value. (2a) Manhattan plot for GWAS in mandarin pulp tissue on chromosomes 1-9 (2b) Quantile-quantile plot for the GWAS in mandarin pulp tissues under MLM. The horizontal axis shows –log10 transformed observed P value, while the vertical axis indicates –log10 transformed expected P value. (3a) Manhattan plot for GWAS in mandarin seed tissue on chromosomes 1-9 (3b) Quantile-quantile plot for the GWAS in mandarin seed tissues under MLM. The horizontal axis shows –log10 transformed observed P value, while the vertical axis indicates –log10 transformed expected P value. The horizontal red line in Manhattan plots indicates a threshold value for significant SNPs. All SNPs with –log10 (P values) greater than 4.9 were considered significant.

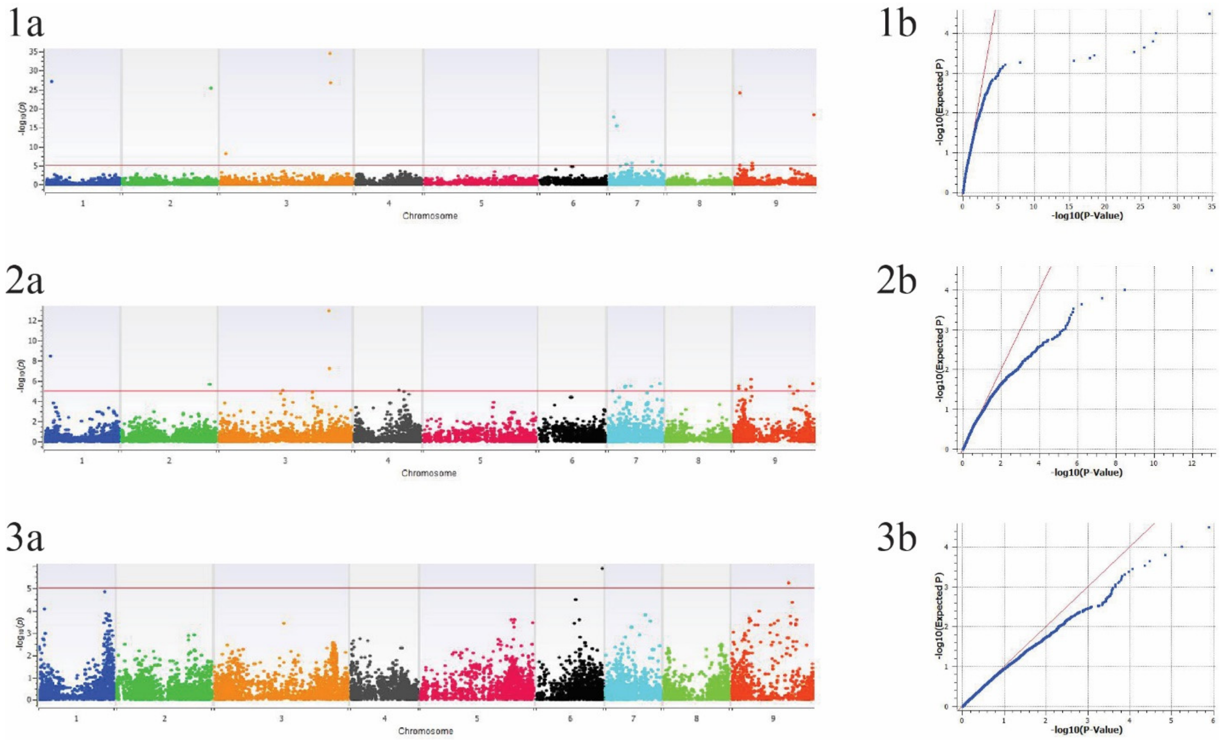

Figure S1-16. Genome wide association analysis of naringin (1a) Manhattan plot for GWAS in mandarin peel tissue on chromosomes 1-9 (1b) Quantile-quantile plot for the GWAS in mandarin peel tissues under MLM. The horizontal axis shows –log10 transformed observed *P* value, while the vertical axis indicates –log10 transformed expected *P* value. (2a) Manhattan plot for GWAS in mandarin pulp tissue on chromosomes 1-9 (2b) Quantile-quantile plot for the GWAS in mandarin pulp tissues under MLM. The horizontal axis shows –log10 transformed observed P value, while the vertical axis indicates –log10 transformed expected P value. (3a) Manhattan plot for GWAS in mandarin seed tissue on chromosomes 1-9 (3b) Quantile-quantile plot for the GWAS in mandarin seed tissues under MLM. The horizontal axis shows –log10 transformed observed P value, while the vertical axis indicates –log10 transformed expected P value. The horizontal red line in Manhattan plots indicates a threshold value for significant SNPs. All SNPs with –log10 (P values) greater than 4.9 were considered significant.

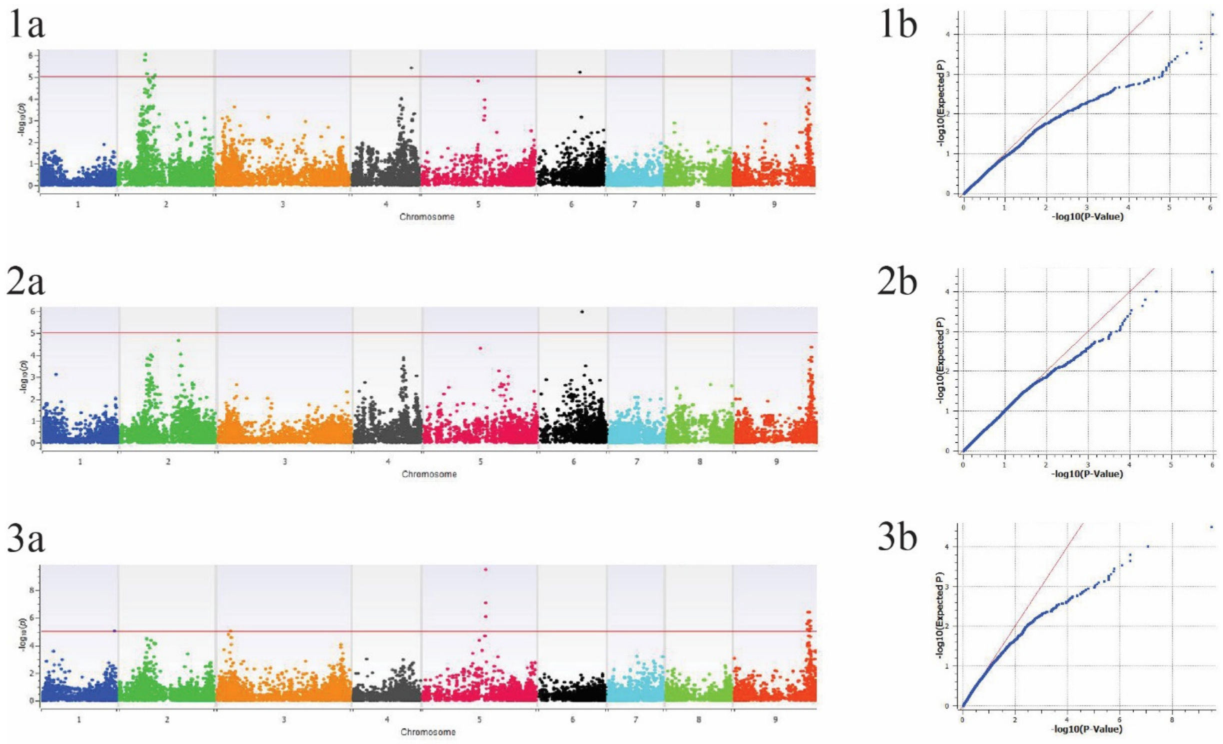

Figure S1-17. Genome wide association analysis of narirutin (1a) Manhattan plot for GWAS in mandarin peel tissue on chromosomes 1-9 (1b) Quantile-quantile plot for the GWAS in mandarin peel tissues under MLM. The horizontal axis shows –log10 transformed observed *P* value, while the vertical axis indicates –log10 transformed expected *P* value. (2a) Manhattan plot for GWAS in mandarin pulp tissue on chromosomes 1-9 (2b) Quantile-quantile plot for the GWAS in mandarin pulp tissues under MLM. The horizontal axis shows –log10 transformed observed P value, while the vertical axis indicates –log10 transformed expected P value. (3a) Manhattan plot for GWAS in mandarin seed tissue on chromosomes 1-9 (3b) Quantile-quantile plot for the GWAS in mandarin seed tissues under MLM. The horizontal axis shows –log10 transformed observed P value, while the vertical axis indicates –log10 transformed expected P value. The horizontal red line in Manhattan plots indicates a threshold value for significant SNPs. All SNPs with –log10 (P values) greater than 4.9 were considered significant.

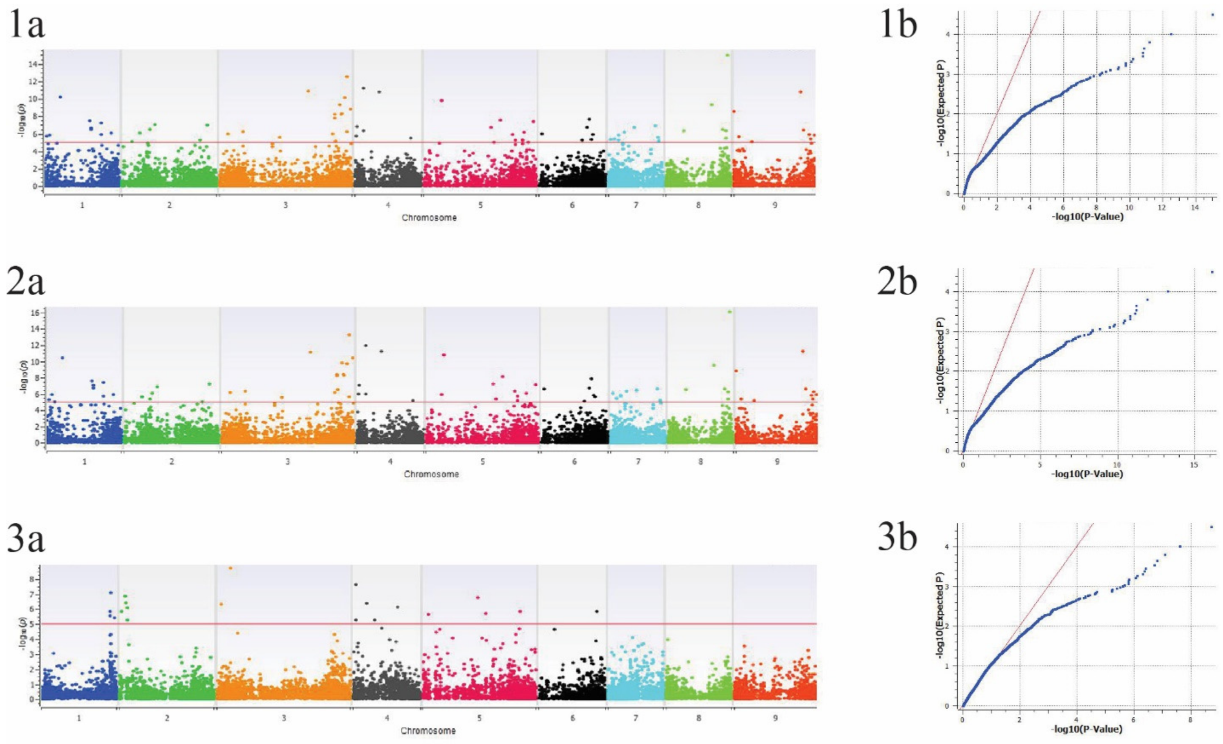

Figure S1-18. Genome wide association analysis of neoeriocitrin (1a) Manhattan plot for GWAS in mandarin peel tissue on chromosomes 1-9 (1b) Quantile-quantile plot for the GWAS in mandarin peel tissues under MLM. The horizontal axis shows –log10 transformed observed *P* value, while the vertical axis indicates –log10 transformed expected *P* value. (2a) Manhattan plot for GWAS in mandarin pulp tissue on chromosomes 1-9 (2b) Quantile-quantile plot for the GWAS in mandarin pulp tissues under MLM. The horizontal axis shows –log10 transformed observed P value, while the vertical axis indicates –log10 transformed expected P value. (3a) Manhattan plot for GWAS in mandarin seed tissue on chromosomes 1-9 (3b) Quantile-quantile plot for the GWAS in mandarin seed tissues under MLM. The horizontal axis shows –log10 transformed observed P value, while the vertical axis indicates –log10 transformed expected P value. The horizontal red line in Manhattan plots indicates a threshold value for significant SNPs. All SNPs with –log10 (P values) greater than 4.9 were considered significant.

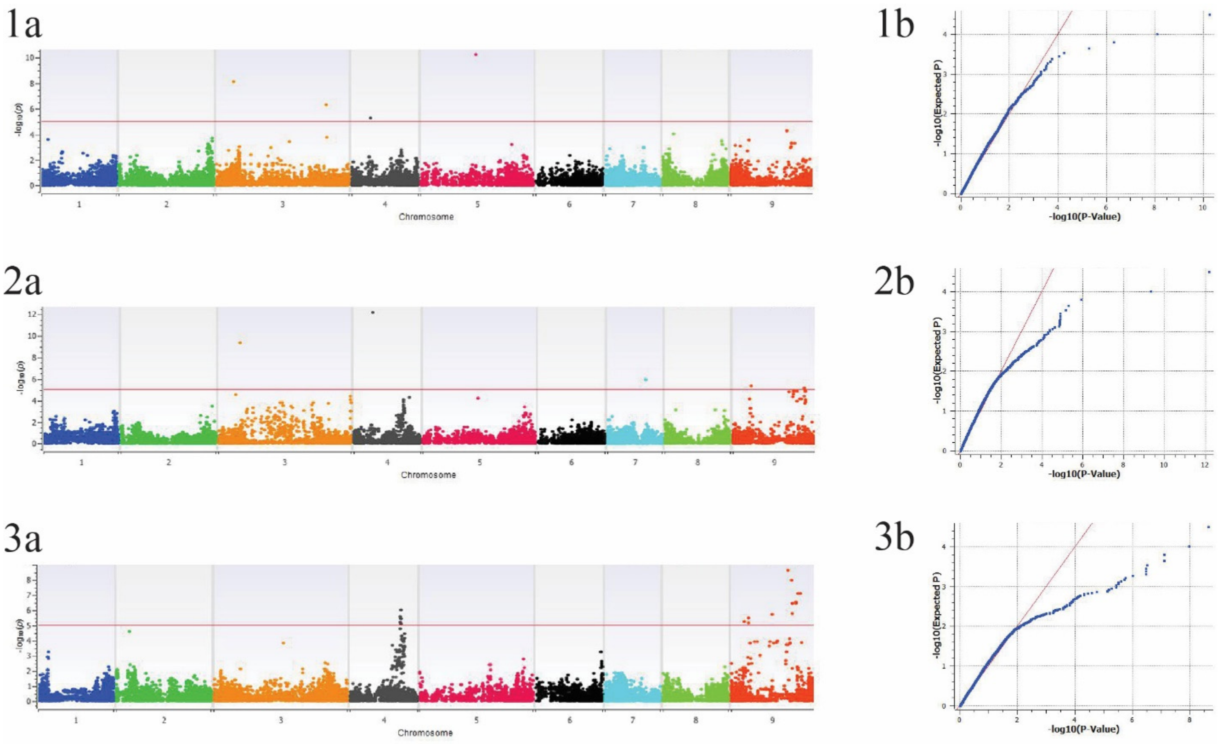

Figure S1-19. Genome wide association analysis of neohesperidin (1a) Manhattan plot for GWAS in mandarin peel tissue on chromosomes 1-9 (1b) Quantile-quantile plot for the GWAS in mandarin peel tissues under MLM. The horizontal axis shows –log10 transformed observed *P* value, while the vertical axis indicates –log10 transformed expected *P* value. (2a) Manhattan plot for GWAS in mandarin pulp tissue on chromosomes 1-9 (2b) Quantile-quantile plot for the GWAS in mandarin pulp tissues under MLM. The horizontal axis shows –log10 transformed observed P value, while the vertical axis indicates –log10 transformed expected P value. (3a) Manhattan plot for GWAS in mandarin seed tissue on chromosomes 1-9 (3b) Quantile-quantile plot for the GWAS in mandarin seed tissues under MLM. The horizontal axis shows – log10 transformed observed P value, while the vertical axis indicates –log10 transformed expected P value. The horizontal red line in Manhattan plots indicates a threshold value for significant SNPs. All SNPs with –log10 (P values) greater than 4.9 were considered significant.

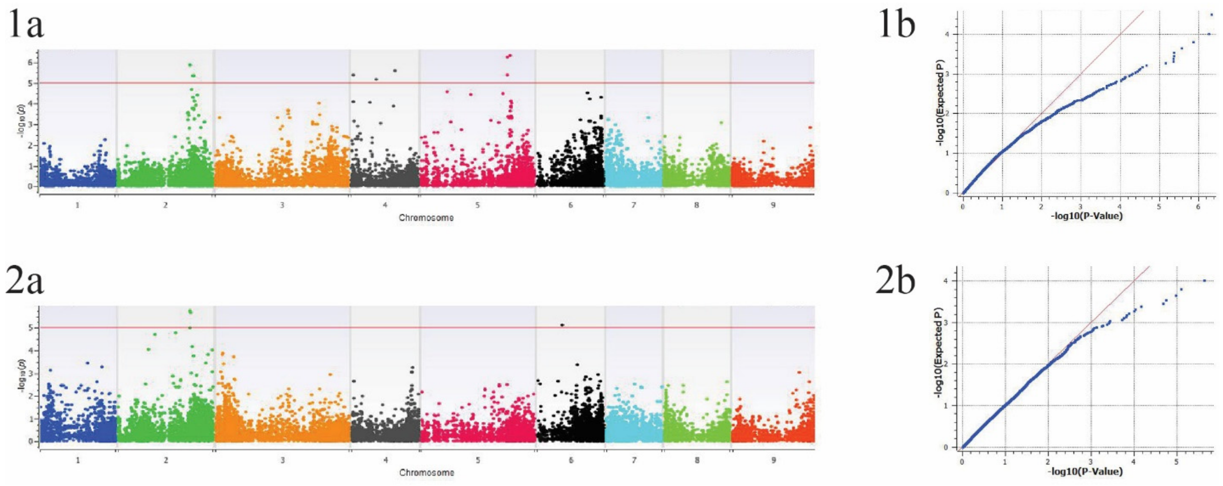

Figure S1-20. Genome wide association analysis of nobiletin (1a) Manhattan plot for GWAS in mandarin pulp tissue on chromosomes 1-9 (1b) Quantile-quantile plot for the GWAS in mandarin pulp tissues under MLM. The horizontal axis shows –log10 transformed observed P value, while the vertical axis indicates –log10 transformed expected P value. (2a) Manhattan plot for GWAS in mandarin seed tissue on chromosomes 1-9 (2b) Quantile-quantile plot for the GWAS in mandarin seed tissues under MLM. The horizontal axis shows –log10 transformed observed P value, while the vertical axis indicates –log10 transformed expected P value. The horizontal red line in Manhattan plots indicates a threshold value for significant SNPs. All SNPs with –log10 (P values) greater than 4.9 were considered significant.

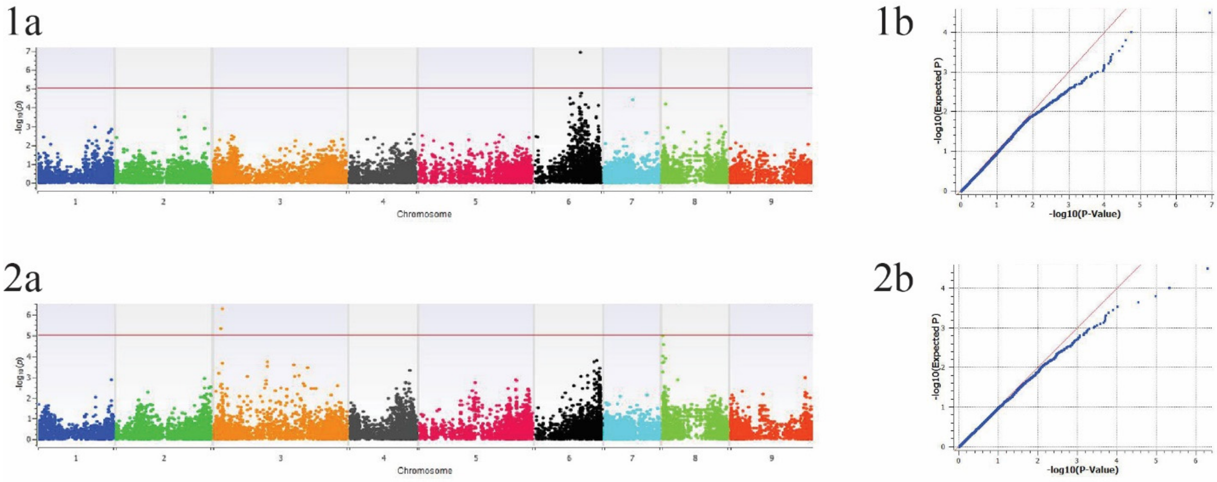

Figure S1-21. Genome wide association analysis of nomilin (1a) Manhattan plot for GWAS in mandarin peel tissue on chromosomes 1-9 (1b) Quantile-quantile plot for the GWAS in mandarin peel tissues under MLM. The horizontal axis shows –log10 transformed observed *P* value, while the vertical axis indicates –log10 transformed expected *P* value. (2a) Manhattan plot for GWAS in mandarin pulp tissue on chromosomes 1-9 (2b) Quantile-quantile plot for the GWAS in mandarin pulp tissues under MLM. The horizontal axis shows –log10 transformed observed P value, while the vertical axis indicates –log10 transformed expected P value. The horizontal red line in Manhattan plots indicates a threshold value for significant SNPs. All SNPs with –log10 (P values) greater than 4.9 were considered significant.

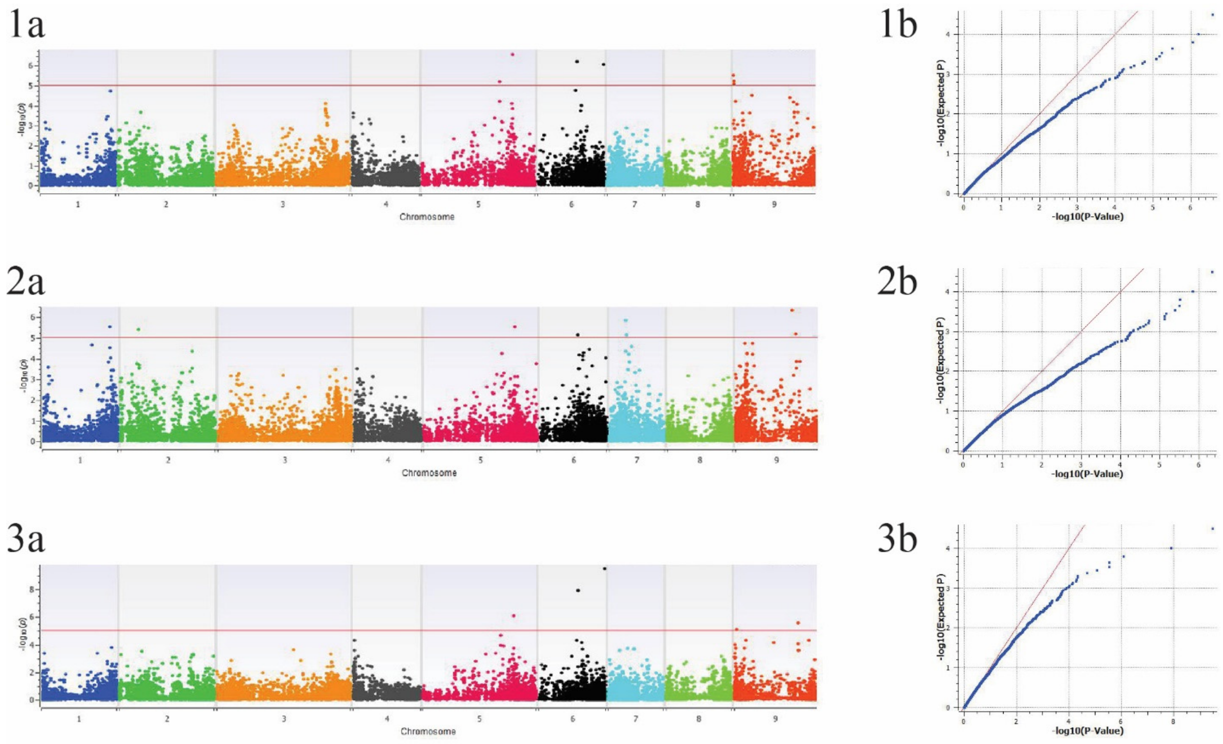

Figure S1-22. Genome wide association analysis of poncirin (1a) Manhattan plot for GWAS in mandarin peel tissue on chromosomes 1-9 (1b) Quantile-quantile plot for the GWAS in mandarin peel tissues under MLM. The horizontal axis shows –log10 transformed observed *P* value, while the vertical axis indicates –log10 transformed expected *P* value. (2a) Manhattan plot for GWAS in mandarin pulp tissue on chromosomes 1-9 (2b) Quantile-quantile plot for the GWAS in mandarin pulp tissues under MLM. The horizontal axis shows –log10 transformed observed P value, while the vertical axis indicates –log10 transformed expected P value. (3a) Manhattan plot for GWAS in mandarin seed tissue on chromosomes 1-9 (3b) Quantile-quantile plot for the GWAS in mandarin seed tissues under MLM. The horizontal axis shows –log10 transformed observed P value, while the vertical axis indicates –log10 transformed expected P value. The horizontal red line in Manhattan plots indicates a threshold value for significant SNPs. All SNPs with –log10 (P values) greater than 4.9 were considered significant.

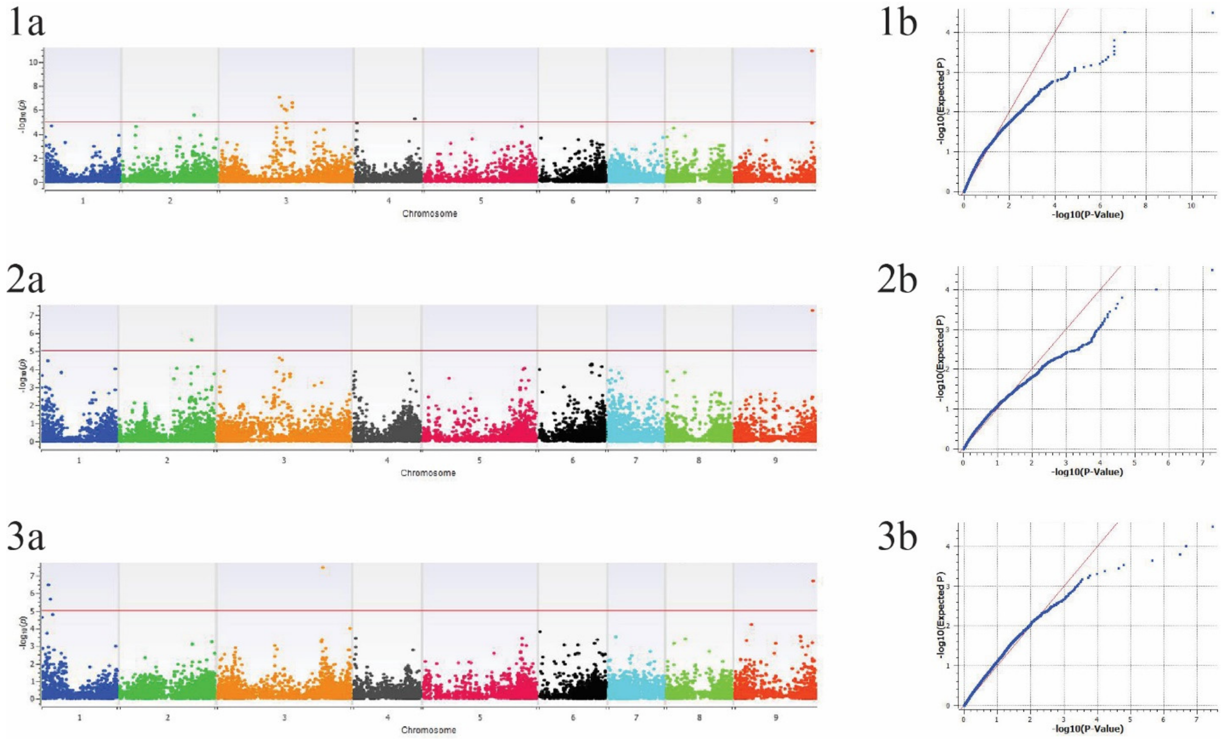

Figure S1-23. Genome wide association analysis of sinensetin (1a) Manhattan plot for GWAS in mandarin peel tissue on chromosomes 1-9 (1b) Quantile-quantile plot for the GWAS in mandarin peel tissues under MLM. The horizontal axis shows –log10 transformed observed *P* value, while the vertical axis indicates –log10 transformed expected *P* value. (2a) Manhattan plot for GWAS in mandarin pulp tissue on chromosomes 1-9 (2b) Quantile-quantile plot for the GWAS in mandarin pulp tissues under MLM. The horizontal axis shows –log10 transformed observed P value, while the vertical axis indicates –log10 transformed expected P value. (3a) Manhattan plot for GWAS in mandarin seed tissue on chromosomes 1-9 (3b) Quantile-quantile plot for the GWAS in mandarin seed tissues under MLM. The horizontal axis shows –log10 transformed observed P value, while the vertical axis indicates –log10 transformed expected P value. The horizontal red line in Manhattan plots indicates a threshold value for significant SNPs. All SNPs with –log10 (P values) greater than 4.9 were considered significant.

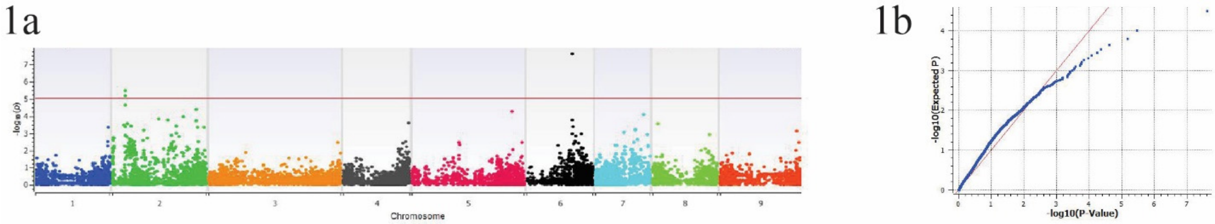

Figure S1-24. Genome wide association analysis of umbelliferone (1a) Manhattan plot for GWAS in mandarin peel tissue on chromosomes 1-9 (1b) Quantile-quantile plot for the GWAS in mandarin peel tissues under MLM. The horizontal axis shows –log10 transformed observed *P* value, while the vertical axis indicates –log10 transformed expected *P* value. The horizontal red line in Manhattan plots indicates a threshold value for significant SNPs. All SNPs with –log10 (P values) greater than 4.9 were considered significant.
