# Supplementary material for: Genome-Wide Association Study of Healthful Flavonoids among Diverse Mandarin Accessions
Source: Plants (Basel). 2022 Jan 25;11(3):317. doi: 10.3390/plants11030317 (PMC8839032; doi:10.3390/plants11030317)
Supplement: Supplementary file 1 [file plants-11-00317-s001.zip › Table S1 Matt's GWAS mandarin ms fg rev.pdf]

Table S1 Diverse mandarin accessions used for the genome wide association of healthful flavonoids

Sample Number	Accession Name	CRC Number^{z}
1	Allspice Tangelo	4143
2	Altoona	2792
3	Amoa 8	4237
4	Austrailian Red	2319
5	Bay Gold	4186
6	Belady	3363
7	Borneo Rangpur	2424
8	Bower	3649
9	*C. amblycarpa*	2485
10	*C. benikojii*	3149
11	*C. depressa*	2448
12	*C. erythrosa* (Fukushu)	3292
13	*C. funadoko*	3274
14	*C. keraji*	3144
15	*C. leiocarpa*	3147
16	*C. nippokoreana*	3228
17	*C. reticulata* (pi433932)	3813
18	*C. reticulata* pi 433931	3812
19	*C. reticulata sdlg.*	3239
20	*C. succosa*	3280
21	*C. sunki*	3143
22	*C. tachibana*	3150
23	*C. tardiva*	3297
24	*C. yatsushiro*	3466
25	Calamondin sdlg.	2592
26	Calashu	2867
27	Canton	3576
28	Chang Sha	3577
29	Clem. Fina	4011
30	Clement	2603
31	Clementine x Pearl	4020
32	Clementine x Silverhill	3731
33	Cleopatra	3844
34	Corsica #1	4046

35	Daisy	3910
36	Dweet	3018
37	Early	2560
38	Emerald Mandarin	4123
39	Fairchild	3559
40	Fallglo (USDA 88-1)	3990
41	Fortune	3560
42	Fremont	3558
43	Frua	3022
44	H-56	3096
45	Hickson	4161
46	Honey	3177
47	Huang Guo Gan #2	rrut103
48	Huang Yen Man Chien	3897
49	Imperial Mandarin	4160
50	Indio	3759
51	Iyo San Ponkan	4118
52	Iyomikan	3980
53	Jamaican Ugli	4127
54	Japansche Citroen	2875
55	Kara	3019
56	Kin Koji Unshiu	3816
57	Kincy	4144
58	King	303
59	King	3845
60	Kinnow	3021
61	Kinnow LS Mandarin	4236
62	Kiyomi H-12	3952
63	Kobayashi Mikan	3817
64	Kunembo	3346
65	Kusaie	452
66	Laranja Cravo	2893
67	Lee	3851
68	Mandarin Sanguine	3367
69	Mandarinette	3405
70	Mapo	4222
71	Marlow Tangelo	4145
72	Mency	3183

73	Michal	4049
74	Minneola	3340
75	Murcott	3846
76	Necked Orange	3738
77	Nissve	3965
78	Nova	3615
79	Novelty x Ellendale	3956
80	Nules	4056
81	Orlando	3874
82	Page	3616
83	Parson’s Special	300
84	Pearl	2849
85	Phillipine	2318
86	Pixie	3568
87	Ponkan	3849
88	Richard’s Special	4117
89	Robinson	3850
90	Rubidoux	4031
91	Ruddy Tangor	4146
92	Sacaton	2609
93	Sampson	2418
94	San Jacinto	2011
95	Santa Barbara	131
96	Satsuma Okitsu-wase	3820
97	Satsuma Owari Frost	3178
98	Scarlet Emperor	3326
99	Seedless Kishu	3906
100	Seminole	2604
101	Sexton	2543
102	Shasta Gold	3973
103	Siamelo	2586
104	Solid Scarlett	3328
105	Som Kaeo	3852
106	Som Keowan	3752
107	SRA 337	4238
108	Sun Chu Sha Kat	4003
109	Sunburst	3809
110	Sunrise	2606

111	Sunshine	2788
112	Szinkom	3085
113	Tahoe Gold	3974
114	Tango Mandarin	4183
115	Tankan	3875
116	TDE1	3972
117	Temple	2598
118	Thorton	2013
119	Tienchen	2590
120	Tim Kat	2692
121	Unnamed Chinese (NGR3294)	ngr3294
122	Unnamed Chinese (NGR3295)	ngr3295
123	USDA 1-77-19	4208
124	USDA 6-15-150	4165
125	USDA 88-2	3991
126	USDA 88-3	3992
127	Varigated Calamondin	3087
128	Webber	2746
129	Wekiwa	2012
130	Wilking	3020
131	Willial Tangelo	4147
132	Williams	2787
133	Willowleaf	3843
134	Yalaha	2559
135	Yellow Rind	3895
136	Yosemite Gold	3975

[z] Inventory and classification number used by the University of California Riverside Citrus Variety Collection to identify acessions
