# Supplementary material for: Genome-Wide Association Study of Healthful Flavonoids among Diverse Mandarin Accessions
Source: Plants (Basel). 2022 Jan 25;11(3):317. doi: 10.3390/plants11030317 (PMC8839032; doi:10.3390/plants11030317)
Supplement: Supplementary file 1 [file plants-11-00317-s001.zip › Table S2 gwas.pdf]

Table S2. List of GWAS determined SNPs in mandarin peel, pulp, and seed tissues

Tissue Type	Compound	Marker	Chromosome	Position	Gene	P-Value
peel	Apigenin	AX-159831918	1	27881393	Ciclev10009180m.g	1.62E-07
peel	Apigenin	AX-160026423	1	28455844	Ciclev10007429m.g	2.72E-07
peel	Apigenin	AX-160641365	1	27906037	Ciclev10009942m.g	6.71E-07
peel	Apigenin	AX-160393747	1	27821623	Ciclev10009640m.g	4.07E-06
peel	Apigenin	AX-159859058	1	28052867	Ciclev10010502m.g	5.89E-06
peel	Apigenin	AX-160750625	1	27150282	Ciclev10007562m.g	5.89E-06
peel	Apigenin	AX-159836698	1	27500224	Ciclev10010082m.g	6.9E-06
peel	Apigenin	AX-159975217	1	27409554	Ciclev10010545m.g	7.27E-06
peel	Apigenin	AX-159909133	1	28153473	Ciclev10007623m.g	7.85E-06
peel	Apigenin	AX-160374383	1	27708189	Ciclev10007594m.g	1E-05
pulp	Apigenin	AX-161017449	3	8373806	Ciclev10019809m.g	1.41E-07
pulp	Apigenin	AX-160815815	7	14849596	Ciclev10025296m.g	4.14E-07
seed	Apigenin	AX-160120038	6	18733554	Ciclev10013344m.g	1.57E-07
seed	Apigenin	AX-159993059	5	17539091	Ciclev10001406m.g	1.1E-06
seed	Apigenin	AX-160440251	6	19982654	Ciclev10011051m.g	6.61E-06
seed	Apigenin	AX-160475114	3	7193544	Ciclev10020967m.g	7.15E-06
seed	Apigenin	AX-160114422	2	31010792	Ciclev10017495m.g	8.06E-06
peel	Didymin	AX-160548920	6	500457	Ciclev10011912m.g	4.94E-08

peel	Didymin	AX-160720530	7	16290180	Ciclev10025265m.g	2.22E-07
peel	Didymin	AX-160475114	3	7193544	Ciclev10020967m.g	2.86E-07
peel	Didymin	AX-160478010	4	23838038	Ciclev10032432m.g	4.65E-07
peel	Didymin	AX-160114422	2	31010792	Ciclev10017495m.g	3.84E-06
peel	Didymin	AX-160821005	2	26517598	Ciclev10014662m.g	4.22E-06
peel	Didymin	AX-160423747	6	17478449	Ciclev10011307m.g	4.32E-06
seed	Didymin	AX-160548920	6	500457	Ciclev10011912m.g	1.69E-08
seed	Didymin	AX-160459025	6	12789826	Ciclev10012329m.g	1.31E-06
seed	Didymin	AX-160720530	7	16290180	Ciclev10025265m.g	6.92E-06
peel	Diosmetin	AX-159831918	1	27881393	Ciclev10009180m.g	1.13E-08
peel	Diosmetin	AX-160379639	3	22843662	Ciclev10023381m.g	7.84E-08
peel	Diosmetin	AX-160026423	1	28455844	Ciclev10007429m.g	4.1E-07
peel	Diosmetin	AX-160641365	1	27906037	Ciclev10009942m.g	6.27E-07
peel	Diosmetin	AX-160934223	1	28785070	Ciclev10008821m.g	1.6E-06
peel	Diosmetin	AX-160055392	1	28404646	Ciclev10007727m.g	1.96E-06
peel	Diosmetin	AX-160138015	1	28434624	Ciclev10008135m.g	2.25E-06
peel	Diosmetin	AX-160393747	1	27821623	Ciclev10009640m.g	4.29E-06
peel	Diosmetin	AX-160750625	1	27150282	Ciclev10007562m.g	5.92E-06
peel	Diosmetin	AX-161062479	3	23750631	Ciclev10024515m.g	6.46E-06

peel	Diosmetin	AX-160100289	3	24900652	Ciclev10020147m.g	7.69E-06
peel	Diosmetin	AX-159975217	1	27409554	Ciclev10010545m.g	8.79E-06
pulp	Diosmetin	AX-160808091	6	23828780	Ciclev10011357m.g	2.99E-06
seed	Diosmetin	AX-160428826	5	34679192	Ciclev10003655m.g	9.47E-20
seed	Diosmetin	AX-159825934	2	3781683	Ciclev10016037m.g	9.37E-19
seed	Diosmetin	AX-161092193	5	39164911	Ciclev10002272m.g	2.19E-15
seed	Diosmetin	AX-160612175	8	1761276	Ciclev10029689m.g	2.45E-14
seed	Diosmetin	AX-160902649	3	2408119	Ciclev10018535m.g	3.99E-13
seed	Diosmetin	AX-160791956	6	23091812	Ciclev10012557m.g	2.05E-12
seed	Diosmetin	AX-160426176	4	21128921	Ciclev10030589m.g	1.39E-09
seed	Diosmetin	AX-160189837	3	33348553	Ciclev10018500m.g	2.11E-09
seed	Diosmetin	AX-160422134	1	28581494	Ciclev10008091m.g	6.01E-09
seed	Diosmetin	AX-160282754	3	43321589	Ciclev10021043m.g	6.87E-08
seed	Diosmetin	AX-160292626	4	641169	Ciclev10031443m.g	6.97E-07
seed	Diosmetin	AX-160397765	8	6060883	Ciclev10030257m.g	9.33E-07
seed	Diosmetin	AX-160759901	5	34902154	Ciclev10002208m.g	1.05E-06
seed	Diosmetin	AX-160781324	1	18689212	Ciclev10008953m.g	2.08E-06
seed	Diosmetin	AX-160197290	1	28376809	Ciclev10007753m.g	2.49E-06
seed	Diosmetin	AX-160299614	8	22899187	Ciclev10027823m.g	3.15E-06

seed	Diosmetin	AX-159861517	7	7306866	Ciclev10026604m.g	4.13E-06
seed	Diosmetin	AX-160278901	1	28375287	Ciclev10007753m.g	4.75E-06
seed	Diosmetin	AX-160882959	1	21332562	Ciclev10009727m.g	4.9E-06
seed	Diosmetin	AX-161004929	8	1427398	Ciclev10028382m.g	6.92E-06
peel	Diosmin	AX-160796087	8	23855395	Ciclev10029047m.g	5.87E-07
peel	Diosmin	AX-160101139	6	19242029	Ciclev10011406m.g	1.39E-06
peel	Diosmin	AX-160647842	5	6741825	Ciclev10003667m.g	5.66E-06
peel	Diosmin	AX-159940685	5	6743512	Ciclev10003667m.g	5.66E-06
peel	Diosmin	AX-160927508	6	18540681	Ciclev10011810m.g	6.59E-06
peel	Diosmin	AX-160875081	2	32880926	Ciclev10016776m.g	8.45E-06
peel	Diosmin	AX-159898445	8	17805422	Ciclev10027942m.g	8.86E-06
pulp	Diosmin	AX-160953115	1	4957396	Ciclev10007412m.g	2.98E-11
pulp	Diosmin	AX-160520944	2	6947881	Ciclev10018398m.g	5.23E-09
pulp	Diosmin	AX-160257746	7	14469316	Ciclev10026727m.g	7.16E-09
pulp	Diosmin	AX-160653457	3	44857767	Ciclev10018517m.g	4.75E-08
pulp	Diosmin	AX-160839831	9	30373460	Ciclev10004580m.g	6.33E-08
pulp	Diosmin	AX-160452448	5	2205630	Ciclev10000456m.g	1.91E-07
pulp	Diosmin	AX-160477116	2	576518	Ciclev10018412m.g	2.5E-07
pulp	Diosmin	AX-160285806	2	6305370	Ciclev10014240m.g	5.04E-07

pulp	Diosmin	AX-160367120	9	3915978	Ciclev10005993m.g	5.3E-07
pulp	Diosmin	AX-160807257	2	5221830	Ciclev10016532m.g	7.13E-07
pulp	Diosmin	AX-160119411	2	5232397	Ciclev10017422m.g	7.13E-07
pulp	Diosmin	AX-160902100	3	47904921	Ciclev10018638m.g	7.25E-07
pulp	Diosmin	AX-159947920	3	47973129	Ciclev10022804m.g	7.25E-07
pulp	Diosmin	AX-160488823	3	48106978	Ciclev10021159m.g	7.25E-07
pulp	Diosmin	AX-160757115	2	8973690	Ciclev10018070m.g	7.98E-07
pulp	Diosmin	AX-159981121	6	19535996	Ciclev10011510m.g	8.15E-07
pulp	Diosmin	AX-160510927	5	34488940	Ciclev10002755m.g	9.33E-07
pulp	Diosmin	AX-161032721	3	31160907	Ciclev10018721m.g	1.44E-06
pulp	Diosmin	AX-160107399	3	1373257	Ciclev10020100m.g	1.47E-06
pulp	Diosmin	AX-160472988	2	5223387	Ciclev10016532m.g	1.98E-06
pulp	Diosmin	AX-160728880	1	18903139	Ciclev10007969m.g	2.17E-06
pulp	Diosmin	AX-160326250	7	10127849	Ciclev10025775m.g	2.32E-06
pulp	Diosmin	AX-160524672	1	19594759	Ciclev10007645m.g	2.85E-06
pulp	Diosmin	AX-160588007	2	6904900	Ciclev10015808m.g	3.04E-06
pulp	Diosmin	AX-160797461	3	48242495	Ciclev10018995m.g	3.57E-06
pulp	Diosmin	AX-160132967	3	48252705	Ciclev10023855m.g	3.57E-06
pulp	Diosmin	AX-160112772	9	30493069	Ciclev10004777m.g	4.64E-06

pulp	Diosmin	AX-160992643	1	27531159	Ciclev10008154m.g	5.32E-06
pulp	Diosmin	AX-160871355	1	10348330	Ciclev10007477m.g	5.7E-06
pulp	Diosmin	AX-160428274	1	16717033	Ciclev10007960m.g	5.7E-06
pulp	Diosmin	AX-160262411	1	26407791	Ciclev10008843m.g	6E-06
pulp	Diosmin	AX-161027264	7	4421541	Ciclev10025134m.g	7.3E-06
pulp	Diosmin	AX-160076955	3	47374838	Ciclev10020049m.g	8E-06
pulp	Diosmin	AX-160494821	1	19312497	Ciclev10010519m.g	8.88E-06
pulp	Diosmin	AX-161071829	7	4447221	Ciclev10027248m.g	9.33E-06
pulp	Diosmin	AX-160517738	5	11468957	Ciclev10002620m.g	1.04E-05
pulp	Diosmin	AX-159893470	9	15158701	Ciclev10004416m.g	1.09E-05
seed	Diosmin	AX-160362613	2	35355786	Ciclev10014319m.g	1.13E-06
seed	Diosmin	AX-160754852	3	29416897	Ciclev10020251m.g	3.83E-06
peel	Eriocitrin	AX-160487850	3	35789790	Ciclev10020864m.g	8.05E-08
peel	Eriocitrin	AX-159902767	4	14477665	Ciclev10030649m.g	4.13E-06
seed	Eriocitrin	AX-160981611	3	5235434	Ciclev10021722m.g	7.55E-10
seed	Eriocitrin	AX-159819588	4	1023454	Ciclev10033546m.g	5.99E-09
seed	Eriocitrin	AX-160938735	2	2367022	Ciclev10018227m.g	7.69E-08
seed	Eriocitrin	AX-160301763	2	3061178	Ciclev10016593m.g	1.77E-07
seed	Eriocitrin	AX-160148759	3	1499963	Ciclev10023031m.g	2.14E-07

seed	Eriocitrin	AX-159948962	4	5291014	Ciclev10032671m.g	2.56E-07
seed	Eriocitrin	AX-160697303	1	26784128	Ciclev10007458m.g	2.87E-07
seed	Eriocitrin	AX-159868580	5	21083453	Ciclev10001079m.g	3.51E-07
seed	Eriocitrin	AX-159959698	2	2472017	Ciclev10017740m.g	5.24E-07
seed	Eriocitrin	AX-159947173	2	896169	Ciclev10015184m.g	5.88E-07
seed	Eriocitrin	AX-160567437	5	2271865	Ciclev10000737m.g	1.17E-06
seed	Eriocitrin	AX-160543749	6	22205333	Ciclev10011364m.g	1.73E-06
seed	Eriocitrin	AX-160599418	4	17018302	Ciclev10031118m.g	1.79E-06
seed	Eriocitrin	AX-160107018	1	28037418	Ciclev10007718m.g	2E-06
seed	Eriocitrin	AX-160463920	5	37150452	Ciclev10000093m.g	2.62E-06
seed	Eriocitrin	AX-160529374	5	24023209	Ciclev10001022m.g	3.15E-06
seed	Eriocitrin	AX-160931127	1	26555666	Ciclev10007305m.g	3.31E-06
seed	Eriocitrin	AX-160728031	2	2984202	Ciclev10016452m.g	3.7E-06
seed	Eriocitrin	AX-160475861	4	8315209	Ciclev10033748m.g	3.7E-06
seed	Eriocitrin	AX-160817184	1	26516346	Ciclev10008581m.g	4.73E-06
seed	Eriocitrin	AX-160933769	4	1172364	Ciclev10033435m.g	4.82E-06
seed	Eriocitrin	AX-160799479	4	10965253	Ciclev10031341m.g	9.98E-06
pulp	Eriodictyol	AX-160808091	6	23828780	Ciclev10011357m.g	2.99E-06
seed	Eriodictyol	AX-160014487	7	1494553	Ciclev10025311m.g	8.81E-10

seed	Eriodictyol	AX-160112361	7	1259780	Ciclev10027377m.g	4.43E-06
pulp	Heptahethoxyflavone	AX-160478010	4	23838038	Ciclev10032432m.g	2.9E-08
pulp	Heptahethoxyflavone	AX-160423747	6	17478449	Ciclev10011307m.g	2.75E-07
pulp	Heptahethoxyflavone	AX-160065656	6	22391215	Ciclev10011395m.g	2.95E-06
pulp	Heptahethoxyflavone	AX-159937988	6	22001439	Ciclev10011719m.g	8.4E-06
seed	Heptahethoxyflavone	AX-159875671	1	23588919	Ciclev10008059m.g	2.91E-07
seed	Heptahethoxyflavone	AX-160792628	2	35342361	Ciclev10018338m.g	3.27E-06
seed	Heptahethoxyflavone	AX-160821005	2	26517598	Ciclev10014662m.g	7.14E-06
seed	Heptahethoxyflavone	AX-160720530	7	16290180	Ciclev10025265m.g	1.08E-05
pulp	Hesperetin	AX-159872199	6	24383951	Ciclev10012224m.g	5.63E-07
seed	Hesperetin	AX-160906983	5	13059166	Ciclev10002443m.g	2.05E-06
pulp	Hesperidin	AX-159823569	7	92972	Ciclev10025023m.g	4.85E-06
pulp	Hesperidin	AX-161076886	7	1183740	Ciclev10024677m.g	8.96E-06
seed	Hesperidin	AX-160261315	5	34690939	Ciclev10002520m.g	1.39E-07
pulp	Isosakurenetin	AX-160865092	3	40172143	Ciclev10024487m.g	1.96E-06
seed	Isosakurenetin	AX-159827950	2	12079122	Ciclev10014123m.g	3.27E-07
seed	Isosakurenetin	AX-159926254	2	12136093	Ciclev10014778m.g	3.27E-07
seed	Isosakurenetin	AX-160365088	3	11421349	Ciclev10022148m.g	1.3E-06
seed	Isosakurenetin	AX-160198906	3	14134551	Ciclev10024059m.g	1.3E-06

seed	Isosakurenetin	AX-160307864	3	18653581	Ciclev10023076m.g	1.3E-06
seed	Isosakurenetin	AX-160191202	3	29985128	Ciclev10018996m.g	1.3E-06
seed	Isosakurenetin	AX-160865092	3	40172143	Ciclev10024487m.g	1.48E-06
seed	Isosakurenetin	AX-160675790	2	11068252	Ciclev10017059m.g	1.61E-06
seed	Isosakurenetin	AX-160865092	3	40172143	Ciclev10024487m.g	1.96E-06
seed	Isosakurenetin	AX-160467531	2	11945984	Ciclev10014114m.g	2.16E-06
seed	Isosakurenetin	AX-160186873	2	12096868	Ciclev10014332m.g	2.61E-06
seed	Isosakurenetin	AX-160298121	2	11131998	Ciclev10018253m.g	3.62E-06
seed	Isosakurenetin	AX-161043592	3	19909499	Ciclev10019664m.g	3.74E-06
seed	Isosakurenetin	AX-160580236	3	32864694	Ciclev10023867m.g	7.35E-06
seed	Isosakurenetin	AX-160551837	2	11383047	Ciclev10017707m.g	7.4E-06
seed	Isosakurenetin	AX-160781332	2	11613704	Ciclev10016580m.g	8.34E-06
seed	Isosakurenetin	AX-160426071	3	43408675	Ciclev10019645m.g	9.11E-06
seed	Isosakurenetin	AX-159935134	3	10502146	Ciclev10020533m.g	1.02E-05
seed	Isosakurenetin	AX-160268972	3	12836580	Ciclev10023517m.g	1.02E-05
seed	Isosakurenetin	AX-160070676	3	18934965	Ciclev10024174m.g	1.02E-05
peel	Isosinensetin	AX-161055987	3	27799655	Ciclev10023071m.g	2.39E-06
peel	Isosinensetin	AX-160548145	3	27804748	Ciclev10019655m.g	2.39E-06
peel	Isosinensetin	AX-160862813	3	27805765	Ciclev10019655m.g	2.39E-06

peel	Isosinensetin	AX-160729031	3	27830592	Ciclev10019217m.g	2.39E-06
peel	Isosinensetin	AX-159862177	3	27851765	Ciclev10023555m.g	3.7E-06
peel	Isosinensetin	AX-161094856	3	25845839	Ciclev10024127m.g	1.15E-05
pulp	Isosinensetin	AX-160432194	2	27643973	Ciclev10014906m.g	9.71E-09
pulp	Isosinensetin	AX-160385265	5	34255060	Ciclev10001494m.g	1.82E-08
pulp	Isosinensetin	AX-160235429	4	802759	Ciclev10031013m.g	2.63E-07
pulp	Isosinensetin	AX-160647029	2	29947323	Ciclev10018411m.g	7.48E-07
pulp	Isosinensetin	AX-160563420	2	30559696	Ciclev10015216m.g	8.68E-07
pulp	Isosinensetin	AX-160014948	5	33018623	Ciclev10000065m.g	1.26E-06
pulp	Isosinensetin	AX-160983298	7	16071299	Ciclev10026360m.g	2.01E-06
pulp	Isosinensetin	AX-161055987	3	27799655	Ciclev10023071m.g	2.16E-06
pulp	Isosinensetin	AX-160548145	3	27804748	Ciclev10019655m.g	2.16E-06
pulp	Isosinensetin	AX-160862813	3	27805765	Ciclev10019655m.g	2.16E-06
pulp	Isosinensetin	AX-160729031	3	27830592	Ciclev10019217m.g	2.16E-06
pulp	Isosinensetin	AX-160398121	2	28980256	Ciclev10017603m.g	3.7E-06
pulp	Isosinensetin	AX-160008695	2	29119709	Ciclev10015906m.g	3.7E-06
pulp	Isosinensetin	AX-159862177	3	27851765	Ciclev10023555m.g	4.47E-06
pulp	Isosinensetin	AX-161062479	3	23750631	Ciclev10024515m.g	4.55E-06
pulp	Isosinensetin	AX-160947519	9	29959571	Ciclev10006155m.g	5.78E-06

pulp	Isosinensetin	AX-160309383	2	28591789	Ciclev10016255m.g	6.87E-06
pulp	Isosinensetin	AX-160423610	5	33179262	Ciclev10001764m.g	9.51E-06
pulp	Isosinensetin	AX-161028752	2	33130969	Ciclev10015688m.g	1.2E-05
seed	Kaempferol	AX-159946295	4	18414143	Ciclev10032158m.g	4.51E-21
seed	Kaempferol	AX-160550404	7	4278015	Ciclev10025483m.g	5.94E-17
seed	Kaempferol	AX-160473016	8	9648249	Ciclev10028552m.g	9.74E-16
seed	Kaempferol	AX-160015982	9	7448706	Ciclev10004188m.g	1.11E-15
seed	Kaempferol	AX-160913504	8	1959240	Ciclev10029403m.g	6.58E-13
seed	Kaempferol	AX-160694677	6	20232781	Ciclev10012009m.g	1.58E-06
seed	Kaempferol	AX-159841151	2	32360356	Ciclev10014504m.g	2.58E-06
seed	Kaempferol	AX-160808091	6	23828780	Ciclev10011357m.g	4.41E-06
peel	Limonin	AX-160548920	6	500457	Ciclev10011912m.g	1.24E-08
peel	Limonin	AX-160423747	6	17478449	Ciclev10011307m.g	3.05E-07
pulp	Limonin	AX-160398164	8	221041	Ciclev10029078m.g	1.79E-06
pulp	Limonin	AX-160423445	8	357915	Ciclev10028672m.g	3.53E-06
pulp	Limonin	AX-160261141	4	22912040	Ciclev10030825m.g	6.44E-06
pulp	Limonin	AX-160127897	4	21278230	Ciclev10033677m.g	8.4E-06
pulp	Limonin	AX-161075964	4	21292618	Ciclev10031713m.g	8.4E-06
pulp	Limonin	AX-160121385	4	21318491	Ciclev10033394m.g	8.4E-06

peel	Luteolin	AX-160742943	6	20232219	Ciclev10012009m.g	1.73E-07
peel	Luteolin	AX-160316365	5	278834	Ciclev10000176m.g	1.51E-06
peel	Luteolin	AX-160557792	6	20636001	Ciclev10012698m.g	2.74E-06
peel	Luteolin	AX-160692119	6	20071784	Ciclev10012467m.g	2.81E-06
pulp	Luteolin	AX-160742943	6	20232219	Ciclev10012009m.g	1.11E-07
pulp	Luteolin	AX-160316365	5	278834	Ciclev10000176m.g	7.79E-07
pulp	Luteolin	AX-160692119	6	20071784	Ciclev10012467m.g	1.39E-06
pulp	Luteolin	AX-160557792	6	20636001	Ciclev10012698m.g	1.63E-06
pulp	Luteolin	AX-160060738	5	31404021	Ciclev10000874m.g	9.84E-06
seed	Luteolin	AX-159825934	2	3781683	Ciclev10016037m.g	1.07E-30
seed	Luteolin	AX-160428826	5	34679192	Ciclev10003655m.g	5.38E-27
seed	Luteolin	AX-161092193	5	39164911	Ciclev10002272m.g	1.36E-22
seed	Luteolin	AX-160902649	3	2408119	Ciclev10018535m.g	1.47E-21
seed	Luteolin	AX-160791956	6	23091812	Ciclev10012557m.g	3.49E-20
seed	Luteolin	AX-159847129	8	23794296	Ciclev10030042m.g	7.37E-20
seed	Luteolin	AX-160422134	1	28581494	Ciclev10008091m.g	3.27E-18
seed	Luteolin	AX-160612175	8	1761276	Ciclev10029689m.g	9.67E-18
seed	Luteolin	AX-160189837	3	33348553	Ciclev10018500m.g	2.48E-15
seed	Luteolin	AX-160461375	4	24910167	Ciclev10031605m.g	2.33E-13

seed	Luteolin	AX-160426176	4	21128921	Ciclev10030589m.g	4.73E-12
seed	Luteolin	AX-160282754	3	43321589	Ciclev10021043m.g	2.62E-08
seed	Luteolin	AX-160461457	4	22361473	Ciclev10033784m.g	2.17E-06
seed	Luteolin	AX-160759901	5	34902154	Ciclev10002208m.g	2.4E-06
peel	Naringenin	AX-160782343	6	19580844	Ciclev10011230m.g	5.13E-08
peel	Naringenin	AX-160808091	6	23828780	Ciclev10011357m.g	7.57E-08
peel	Naringenin	AX-160310225	6	19678458	Ciclev10013089m.g	8.27E-07
peel	Naringenin	AX-160549437	6	17263467	Ciclev10012677m.g	1.66E-06
peel	Naringenin	AX-159981121	6	19535996	Ciclev10011510m.g	4.73E-06
peel	Naringenin	AX-160401921	6	15140598	Ciclev10012647m.g	9.99E-06
peel	Naringenin	AX-159927591	6	16822576	Ciclev10011992m.g	9.99E-06
pulp	Naringenin	AX-160808091	6	23828780	Ciclev10011357m.g	2.49E-07
seed	Naringenin	AX-159951409	5	24061380	Ciclev10001582m.g	2.38E-07
seed	Naringenin	AX-160444742	3	34314371	Ciclev10019630m.g	1.06E-06
seed	Naringenin	AX-160736569	4	22753135	Ciclev10031081m.g	1.22E-06
seed	Naringenin	AX-160455793	5	21522005	Ciclev10001212m.g	1.43E-06
seed	Naringenin	AX-160364658	2	34502934	Ciclev10014430m.g	1.7E-06
seed	Naringenin	AX-161035795	5	24022193	Ciclev10001022m.g	2.34E-06
seed	Naringenin	AX-160798069	3	4493879	Ciclev10020548m.g	3.93E-06

seed	Naringenin	AX-160247134	5	23726612	Ciclev10000349m.g	4.24E-06
seed	Naringenin	AX-159827950	2	12079122	Ciclev10014123m.g	4.9E-06
seed	Naringenin	AX-159926254	2	12136093	Ciclev10014778m.g	4.9E-06
seed	Naringenin	AX-159876092	2	10245424	Ciclev10018210m.g	5.17E-06
seed	Naringenin	AX-160871858	2	10250942	Ciclev10016536m.g	5.17E-06
seed	Naringenin	AX-159907005	2	13961951	Ciclev10014385m.g	7.14E-06
seed	Naringenin	AX-160215597	2	33285994	Ciclev10014912m.g	7.19E-06
seed	Naringenin	AX-160635634	2	34278244	Ciclev10015080m.g	7.19E-06
seed	Naringenin	AX-160653545	2	34528273	Ciclev10015910m.g	7.19E-06
seed	Naringenin	AX-159984792	3	9278465	Ciclev10024246m.g	9.35E-06
seed	Naringenin	AX-161026143	2	34869109	Ciclev10014824m.g	1.14E-05
seed	Naringenin	AX-161053191	2	34879365	Ciclev10015513m.g	1.14E-05
pulp	Naringin	AX-160259155	3	42212133	Ciclev10022115m.g	1.02E-13
pulp	Naringin	AX-160267330	1	2747213	Ciclev10007564m.g	3.63E-09
pulp	Naringin	AX-159836993	3	42505301	Ciclev10019202m.g	5.58E-08
pulp	Naringin	AX-160248844	9	6696818	Ciclev10006216m.g	6.34E-07
pulp	Naringin	AX-159957846	7	20069163	Ciclev10027591m.g	1.8E-06
pulp	Naringin	AX-160336963	9	30375702	Ciclev10005967m.g	1.85E-06
pulp	Naringin	AX-159996057	2	33843185	Ciclev10017311m.g	2.29E-06

pulp	Naringin	AX-159921993	7	6612534	Ciclev10027602m.g	2.83E-06
pulp	Naringin	AX-160919322	9	2056652	Ciclev10004220m.g	2.84E-06
pulp	Naringin	AX-160725324	7	8638275	Ciclev10027587m.g	2.9E-06
pulp	Naringin	AX-160086540	7	16602937	Ciclev10025204m.g	3.46E-06
pulp	Naringin	AX-160395823	9	21603308	Ciclev10005135m.g	3.64E-06
pulp	Naringin	AX-160961265	7	6409738	Ciclev10026255m.g	4.37E-06
pulp	Naringin	AX-161027615	9	6700428	Ciclev10005943m.g	4.4E-06
pulp	Naringin	AX-160539441	9	6700557	Ciclev10005943m.g	4.4E-06
pulp	Naringin	AX-160095131	9	2112575	Ciclev10004654m.g	5.8E-06
pulp	Naringin	AX-160633921	9	4925460	Ciclev10004796m.g	7.15E-06
pulp	Naringin	AX-160849709	4	17173787	Ciclev10031611m.g	8.51E-06
pulp	Naringin	AX-160908535	3	24746384	Ciclev10019165m.g	8.51E-06
pulp	Naringin	AX-160997029	9	24660983	Ciclev10005512m.g	8.6E-06
pulp	Naringin	AX-160798466	7	1808377	Ciclev10025723m.g	9.08E-06
pulp	Naringin	AX-160157950	4	19247937	Ciclev10031332m.g	1.18E-05
pulp	Naringin	AX-161023640	3	35986885	Ciclev10018708m.g	1.21E-05
seed	Naringin	AX-159969067	6	25181051	Ciclev10011245m.g	1.3E-06
seed	Naringin	AX-160395823	9	21603308	Ciclev10005135m.g	5.79E-06
peel	Narirutin	AX-160169667	6	16177024	Ciclev10011991m.g	1.07E-06

pulp	Narirutin	AX-160771302	2	10404320	Ciclev10015266m.g	9.22E-07
pulp	Narirutin	AX-160754042	2	10537608	Ciclev10015043m.g	9.22E-07
pulp	Narirutin	AX-159876092	2	10245424	Ciclev10018210m.g	1.79E-06
pulp	Narirutin	AX-160871858	2	10250942	Ciclev10016536m.g	1.79E-06
pulp	Narirutin	AX-160736569	4	22753135	Ciclev10031081m.g	3.99E-06
pulp	Narirutin	AX-160169667	6	16177024	Ciclev10011991m.g	6.59E-06
pulp	Narirutin	AX-160675790	2	11068252	Ciclev10017059m.g	7.57E-06
pulp	Narirutin	AX-159907005	2	13961951	Ciclev10014385m.g	8.98E-06
pulp	Narirutin	AX-159853043	2	13244381	Ciclev10015977m.g	1.07E-05
pulp	Narirutin	AX-160602311	2	13478860	Ciclev10014073m.g	1.07E-05
seed	Narirutin	AX-160529374	5	24023209	Ciclev10001022m.g	3.28E-10
seed	Narirutin	AX-159951409	5	24061380	Ciclev10001582m.g	8.96E-08
seed	Narirutin	AX-160340760	9	28267616	Ciclev10005338m.g	4.1E-07
seed	Narirutin	AX-160629786	9	28803668	Ciclev10004647m.g	4.1E-07
seed	Narirutin	AX-161035795	5	24022193	Ciclev10001022m.g	8.83E-07
seed	Narirutin	AX-160321416	9	28554807	Ciclev10004825m.g	1.68E-06
seed	Narirutin	AX-160255940	9	29053060	Ciclev10006075m.g	1.79E-06
seed	Narirutin	AX-160409880	9	28402296	Ciclev10005677m.g	2.28E-06
seed	Narirutin	AX-160708797	9	27726456	Ciclev10004462m.g	2.73E-06

seed	Narirutin	AX-160550656	9	27941277	Ciclev10005783m.g	2.73E-06
seed	Narirutin	AX-160769450	9	27965072	Ciclev10004147m.g	2.73E-06
seed	Narirutin	AX-160185021	9	29015985	Ciclev10004124m.g	4.03E-06
seed	Narirutin	AX-159887419	9	28967403	Ciclev10007061m.g	6.67E-06
seed	Narirutin	AX-160633598	9	28074494	Ciclev10004987m.g	7.61E-06
seed	Narirutin	AX-160173746	3	5262538	Ciclev10018727m.g	8.82E-06
seed	Narirutin	AX-160107018	1	28037418	Ciclev10007718m.g	9.35E-06
seed	Narirutin	AX-161030870	9	28765403	Ciclev10004900m.g	9.74E-06
seed	Neoeriocitrin	AX-160981611	3	5235434	Ciclev10021722m.g	1.89E-09
seed	Neoeriocitrin	AX-159819588	4	1023454	Ciclev10033546m.g	2.45E-08
seed	Neoeriocitrin	AX-160697303	1	26784128	Ciclev10007458m.g	8.28E-08
seed	Neoeriocitrin	AX-160938735	2	2367022	Ciclev10018227m.g	1.52E-07
seed	Neoeriocitrin	AX-159868580	5	21083453	Ciclev10001079m.g	1.89E-07
seed	Neoeriocitrin	AX-159959698	2	2472017	Ciclev10017740m.g	3.92E-07
seed	Neoeriocitrin	AX-159948962	4	5291014	Ciclev10032671m.g	4.28E-07
seed	Neoeriocitrin	AX-160148759	3	1499963	Ciclev10023031m.g	4.9E-07
seed	Neoeriocitrin	AX-160599418	4	17018302	Ciclev10031118m.g	7.55E-07
seed	Neoeriocitrin	AX-160301763	2	3061178	Ciclev10016593m.g	8.82E-07
seed	Neoeriocitrin	AX-160463920	5	37150452	Ciclev10000093m.g	1.48E-06

seed	Neoeriocitrin	AX-160817184	1	26516346	Ciclev10008581m.g	1.53E-06
seed	Neoeriocitrin	AX-159947173	2	896169	Ciclev10015184m.g	1.54E-06
seed	Neoeriocitrin	AX-160543749	6	22205333	Ciclev10011364m.g	1.56E-06
seed	Neoeriocitrin	AX-160529374	5	24023209	Ciclev10001022m.g	2.12E-06
seed	Neoeriocitrin	AX-160567437	5	2271865	Ciclev10000737m.g	2.43E-06
seed	Neoeriocitrin	AX-160931127	1	26555666	Ciclev10007305m.g	2.97E-06
seed	Neoeriocitrin	AX-160107018	1	28037418	Ciclev10007718m.g	4.05E-06
seed	Neoeriocitrin	AX-160933769	4	1172364	Ciclev10033435m.g	5.62E-06
seed	Neoeriocitrin	AX-160728031	2	2984202	Ciclev10016452m.g	5.87E-06
seed	Neoeriocitrin	AX-160475861	4	8315209	Ciclev10033748m.g	5.87E-06
peel	Neohesperidin	AX-159868580	5	21083453	Ciclev10001079m.g	5.47E-11
peel	Neohesperidin	AX-160079311	3	6562328	Ciclev10024422m.g	8.32E-09
peel	Neohesperidin	AX-160259155	3	42212133	Ciclev10022115m.g	5.02E-07
peel	Neohesperidin	AX-160292039	4	7374156	Ciclev10033003m.g	5.21E-06
pulp	Neohesperidin	AX-160292039	4	7374156	Ciclev10033003m.g	6.81E-13
pulp	Neohesperidin	AX-161017449	3	8373806	Ciclev10019809m.g	4.65E-10
pulp	Neohesperidin	AX-160815815	7	14849596	Ciclev10025296m.g	1.2E-06
pulp	Neohesperidin	AX-160811575	9	7080103	Ciclev10004776m.g	5.14E-06
pulp	Neohesperidin	AX-160545680	9	27487565	Ciclev10006597m.g	7.15E-06

seed	Neohesperidin	AX-160395823	9	21603308	Ciclev10005135m.g	2.29E-09
seed	Neohesperidin	AX-160748919	9	23071013	Ciclev10005494m.g	1.07E-08
seed	Neohesperidin	AX-160518070	9	25644906	Ciclev10006760m.g	7.87E-08
seed	Neohesperidin	AX-160906318	9	26260000	Ciclev10006703m.g	7.87E-08
seed	Neohesperidin	AX-160997029	9	24660983	Ciclev10005512m.g	3.12E-07
seed	Neohesperidin	AX-160153520	9	23415914	Ciclev10004128m.g	3.5E-07
seed	Neohesperidin	AX-160733435	9	23451840	Ciclev10004347m.g	3.5E-07
seed	Neohesperidin	AX-160287356	9	24649589	Ciclev10007128m.g	3.5E-07
seed	Neohesperidin	AX-159899984	4	19530036	Ciclev10031441m.g	9.94E-07
seed	Neohesperidin	AX-160724120	9	23227951	Ciclev10005698m.g	1.75E-06
seed	Neohesperidin	AX-160648709	9	15655218	Ciclev10006483m.g	1.93E-06
seed	Neohesperidin	AX-160157950	4	19247937	Ciclev10031332m.g	2.56E-06
seed	Neohesperidin	AX-161027615	9	6700428	Ciclev10005943m.g	3.21E-06
seed	Neohesperidin	AX-160539441	9	6700557	Ciclev10005943m.g	3.21E-06
seed	Neohesperidin	AX-160036418	4	19448701	Ciclev10030630m.g	3.73E-06
seed	Neohesperidin	AX-160469036	4	19476477	Ciclev10031047m.g	3.73E-06
seed	Neohesperidin	AX-160699905	4	19480477	Ciclev10031047m.g	3.73E-06
seed	Neohesperidin	AX-160633921	9	4925460	Ciclev10004796m.g	5.37E-06
seed	Neohesperidin	AX-160334519	4	19213869	Ciclev10031861m.g	6.55E-06

seed	Neohesperidin	AX-160248844	9	6696818	Ciclev10006216m.g	6.87E-06
seed	Neohesperidin	AX-160271632	4	19472168	Ciclev10031089m.g	7.47E-06
pulp	Nobiletin	AX-160385265	5	34255060	Ciclev10001494m.g	4.72E-07
pulp	Nobiletin	AX-160014948	5	33018623	Ciclev10000065m.g	5.61E-07
pulp	Nobiletin	AX-160432194	2	27643973	Ciclev10014906m.g	1.36E-06
pulp	Nobiletin	AX-160502138	4	16736881	Ciclev10031340m.g	2.73E-06
pulp	Nobiletin	AX-160235429	4	802759	Ciclev10031013m.g	4.25E-06
pulp	Nobiletin	AX-160423610	5	33179262	Ciclev10001764m.g	4.26E-06
pulp	Nobiletin	AX-160398121	2	28980256	Ciclev10017603m.g	4.45E-06
pulp	Nobiletin	AX-160008695	2	29119709	Ciclev10015906m.g	4.45E-06
pulp	Nobiletin	AX-160298355	4	9496767	Ciclev10033763m.g	6.95E-06
seed	Nobiletin	AX-160079410	2	27608690	Ciclev10017596m.g	1.77E-06
seed	Nobiletin	AX-160432194	2	27643973	Ciclev10014906m.g	2.27E-06
seed	Nobiletin	AX-160545039	6	9677503	Ciclev10011376m.g	8E-06
seed	Nobiletin	AX-160728913	2	27612460	Ciclev10014623m.g	1.07E-05
peel	Nomilin	AX-160423747	6	17478449	Ciclev10011307m.g	1.19E-07
pulp	Nomilin	AX-160298860	3	3171806	Ciclev10022532m.g	5.22E-07
pulp	Nomilin	AX-160290069	3	2599353	Ciclev10020622m.g	4.84E-06
peel	Poncirin	AX-160261315	5	34690939	Ciclev10002520m.g	2.68E-07

peel	Poncirin	AX-160446122	6	15225342	Ciclev10013024m.g	6.5E-07
peel	Poncirin	AX-159969067	6	25181051	Ciclev10011245m.g	8.92E-07
peel	Poncirin	AX-160123365	9	48393	Ciclev10005749m.g	3.17E-06
peel	Poncirin	AX-160280996	9	194972	Ciclev10005631m.g	5.99E-06
peel	Poncirin	AX-160885377	5	29852999	Ciclev10003382m.g	6.78E-06
peel	Poncirin	AX-160929088	9	178813	Ciclev10005819m.g	8.28E-06
pulp	Poncirin	AX-160395823	9	21603308	Ciclev10005135m.g	4.68E-07
pulp	Poncirin	AX-160961265	7	6409738	Ciclev10026255m.g	1.46E-06
pulp	Poncirin	AX-160467463	1	25802816	Ciclev10007519m.g	3.13E-06
pulp	Poncirin	AX-160261315	5	34690939	Ciclev10002520m.g	3.17E-06
pulp	Poncirin	AX-160380538	2	6823063	Ciclev10017658m.g	4.06E-06
pulp	Poncirin	AX-160748919	9	23071013	Ciclev10005494m.g	6.98E-06
pulp	Poncirin	AX-159921993	7	6612534	Ciclev10027602m.g	7.53E-06
pulp	Poncirin	AX-160709017	6	14529529	Ciclev10011508m.g	7.65E-06
seed	Poncirin	AX-159969067	6	25181051	Ciclev10011245m.g	3.39E-10
seed	Poncirin	AX-160446122	6	15225342	Ciclev10013024m.g	1.27E-08
seed	Poncirin	AX-160261315	5	34690939	Ciclev10002520m.g	8.46E-07
seed	Poncirin	AX-160532763	9	24335382	Ciclev10006928m.g	2.95E-06
seed	Poncirin	AX-159832230	9	24347602	Ciclev10006928m.g	2.95E-06

seed	Poncirin	AX-160157742	9	887430	Ciclev10004497m.g	8.51E-06
peel	Sinensetin	AX-160947519	9	29959571	Ciclev10006155m.g	1.25E-11
peel	Sinensetin	AX-160379639	3	22843662	Ciclev10023381m.g	8.94E-08
peel	Sinensetin	AX-161055987	3	27799655	Ciclev10023071m.g	2.68E-07
peel	Sinensetin	AX-160548145	3	27804748	Ciclev10019655m.g	2.68E-07
peel	Sinensetin	AX-160862813	3	27805765	Ciclev10019655m.g	2.68E-07
peel	Sinensetin	AX-160729031	3	27830592	Ciclev10019217m.g	2.68E-07
peel	Sinensetin	AX-161062479	3	23750631	Ciclev10024515m.g	4.83E-07
peel	Sinensetin	AX-159862177	3	27851765	Ciclev10023555m.g	5.97E-07
peel	Sinensetin	AX-160100289	3	24900652	Ciclev10020147m.g	8.38E-07
peel	Sinensetin	AX-161094856	3	25845839	Ciclev10024127m.g	1.1E-06
peel	Sinensetin	AX-160432194	2	27643973	Ciclev10014906m.g	2.78E-06
peel	Sinensetin	AX-160967845	4	22863590	Ciclev10031448m.g	5.66E-06
pulp	Sinensetin	AX-160947519	9	29959571	Ciclev10006155m.g	5.58E-08
pulp	Sinensetin	AX-160432194	2	27643973	Ciclev10014906m.g	2.4E-06
seed	Sinensetin	AX-160284313	3	40137702	Ciclev10020460m.g	3.48E-08
seed	Sinensetin	AX-160947519	9	29959571	Ciclev10006155m.g	2.18E-07
seed	Sinensetin	AX-160420792	1	2684250	Ciclev10007225m.g	3.35E-07
seed	Sinensetin	AX-160161470	1	3428750	Ciclev10010686m.g	2.21E-06

peel	Umbelliferone	AX-160324465	6	17484352	Ciclev10011044m.g	2.5E-08
peel	Umbelliferone	AX-160360188	2	5017716	Ciclev10015088m.g	3.51E-06
peel	Umbelliferone	AX-159951546	2	5021619	Ciclev10014066m.g	6.78E-06
